# Osteopontin promotes metastasis of intrahepatic cholangiocarcinoma through recruiting MAPK1 and mediating Ser675 phosphorylation of β-Catenin

**DOI:** 10.1038/s41419-017-0226-x

**Published:** 2018-02-07

**Authors:** Yan Zheng, Chuang Zhou, Xin-Xin Yu, Chao Wu, Hu-Liang Jia, Xiao-Mei Gao, Ji-Meng Yang, Chao-Qun Wang, Qin Luo, Ying Zhu, Yu Zhang, Jin-Wang Wei, Yuan-Yuan Sheng, Qiong-Zhu Dong, Lun-Xiu Qin

**Affiliations:** Department of General Surgery, Huashan Hospital & Cancer Metastasis Institute & Institutes of Biomedical Sciences, Fudan University, Shanghai, China

## Abstract

The incidence and mortality of intrahepatic cholangiocarcinoma (ICC) are increasing worldwide in recent decades. Osteopontin (OPN) plays an important role in cancer metastasis, but its functional mechanism in ICC is not clear yet. In this study, we found that OPN level was elevated both in plasma and tumor tissues of ICC patients, which was closely related to a shorter overall survival (OS) and high probability of tumor relapse after curative resection. The gain- and loss-of-function studies determined that OPN could promote ICC growth and metastasis. OPN selectively interacted with β-Catenin and knockdown of β-Catenin abrogated the effects induced by OPN. OPN recruited MAPK1 and activated MEK–MAPK1 pathway to mediate the S675 phosphorylation of β-Catenin and nucleus accumulation, which induced the activation of Wnt signaling. Moreover, a significant correlation between OPN and β-Catenin was found in ICC tissues. OPN, β-Catenin, and their combination were independent prognostic indicator for ICC patients. In conclusion, OPN promotes ICC progression through recruiting MAPK1 and activating the Wnt/β-Catenin pathway and can serve as a novel prognostic marker and therapeutic target for ICC.

## Introduction

Intrahepatic cholangiocarcinoma (ICC), arising from the intrahepatic bile ducts, is the second most common primary liver cancer^1,2^. In recent decades, the incidence and mortality rate of ICC have been increasing in the world^3^. ICC is characterized as a devastating disease with very dismal prognosis^4^. Currently, surgical resection and liver transplantation are the main treatment options; however, the 5-year survival rate is still very low due to the high probability of metastasis and relapse^5,6^. The pathogenesis of ICC is very complicated. Several signaling pathway deregulations that are related to the inflammatory, stress response, tumor growth, and metastasis have been reported to be involved, such as JAK–IL-6-STAT3, EGFR and HGF/MET, RAS/MAPK, NOTCH and WNT signaling^4,6,7^. Unfortunately, to date no molecular targeted therapy is effective for ICC^6^. Therefore, it is urgent to define the molecular mechanism involved in the progression of ICC.

Osteopontin (OPN) is a chemokine-like phosphorylated glycoprotein, remaining intracellular or secreted, which is frequently upregulated in numerous human cancers and plays a pivotal role in proliferation, stemness, inflammatory response, extracellular matrix (ECM) degradation, angiogenesis, invasion, and metastasis^8–11^. In our previous studies, we have confirmed that OPN plays important roles in promoting metastasis of hepatocellular carcinoma (HCC)^12–15^. Recent evidence from other studies showed that OPN was highly expressed in the tumor and stroma of ICC tissues^16,17^. Moreover, its expression in the stroma was closely associated with the overall survival (OS) of ICC patients^17^. However, little is understood about the function role and mechanism of OPN in ICC growth and metastasis.

In the present study, we found that OPN was over-expressed in ICC tissues, which was closely associated with worse clinical outcomes and played a crucial role in tumor growth and metastasis. OPN directly interacted with β-Catenin and mediated its S675 phosphorylation and nucleus translocation by recruiting and activating MAPK1, which could upregulate Wnt target genes *C-MYC*, *CYCLIN-D1*, and *PROX1*. These results provide a better understanding of the underlying mechanism by which OPN involved in the progression and metastasis of ICC.

## Results

### High OPN expression is associated with dismal outcomes of patients with ICC

**Fig. 1:**
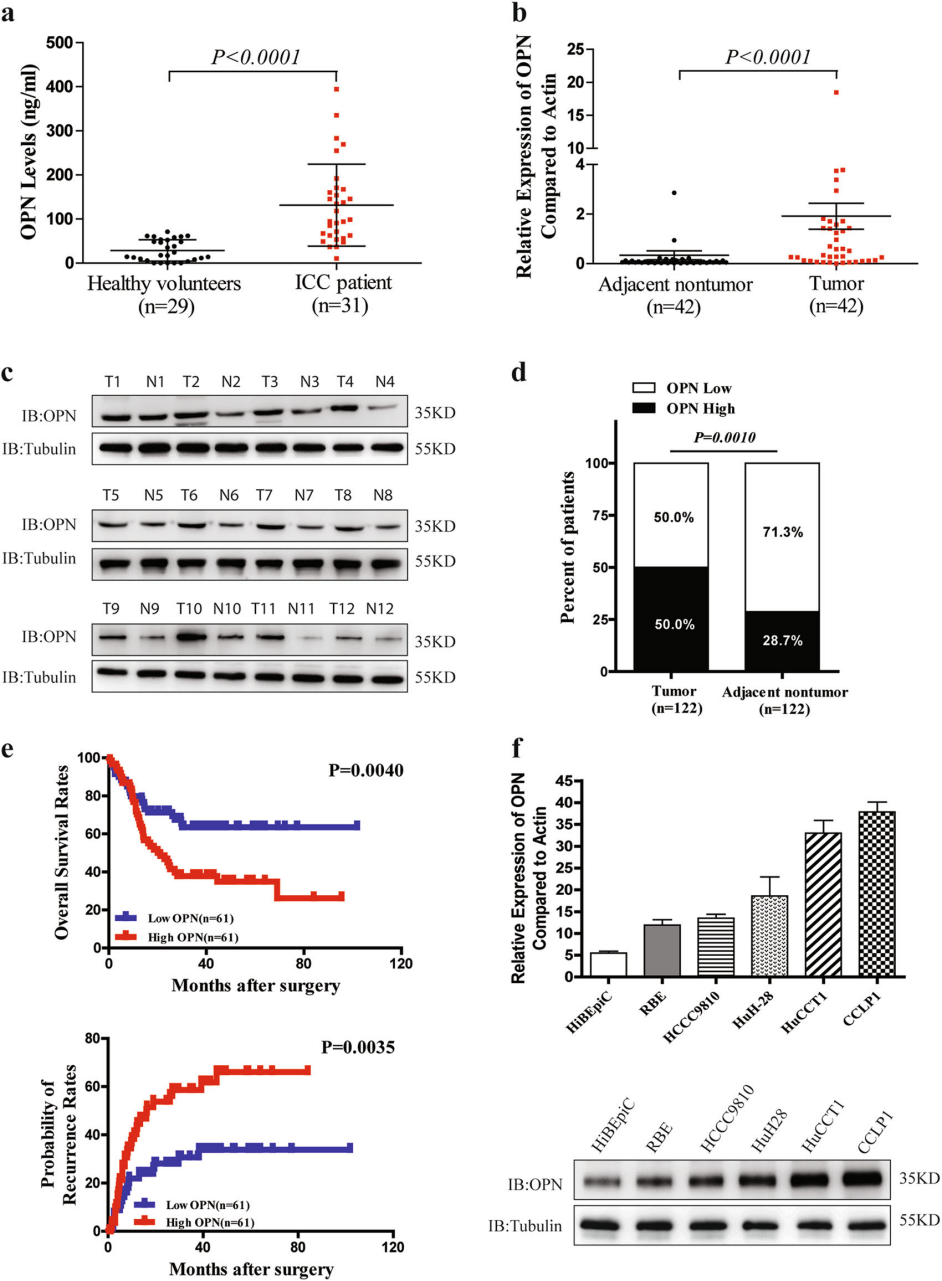
OPN expression level is closely associated with metastasis and recurrence of ICC. **a** Preoperative plasma OPN levels of ICC patients and healthy volunteers were assessed by ELISA. **b** Relative expression of OPN mRNA in ICC tumor and adjacent non-tumor liver tissues was analyzed by qRT-PCR. **c** OPN protein levels were detected by western blot in 12 pairs of frozen ICC tissues, T tumors; N adjacent non-tumor tissues. **d** IHC analysis of OPN expression in 122 ICC tumor and adjacent non-tumor liver tissues. **e** OS rates and probabilities of tumor recurrence in ICC patients with low or high OPN expression assessed by Kaplan–Meier analysis. **f** qRT-PCR and western blot were performed to determine OPN expression level in HiBEpiC and ICC cell lines controlled by β-actin and β-tubulin

The plasma OPN levels of ICC patients were remarkably increased compared with the healthy volunteers detected by enzyme-linked immunosorbent assay (ELISA) (Fig. 1a). And quantitative reverse transcriptase polymerase chain reaction (qRT-PCR) assays also showed a significant up-regulation of OPN mRNA expression in ICC tissues compared with their matched adjacent non-tumor liver tissues (Fig. 1b). These were further verified in protein level using western blot and immunohistochemistry (IHC) (Fig. 1c, d). Furthermore, OPN expression was found to be significantly associated with γ-glutamyl transpeptadase level (GGT), TNM stage, and particularly with the regional lymph node metastasis (Supplementary Table 1). Using the median OPN levels as the cutoff value, the patients in the low-OPN group had significantly better OS and lower possibilities of tumor recurrence than those in the high-OPN group (Fig. 1e). Univariate analysis showed that OPN expression, regional lymph node metastasis, and tumor differentiation were significantly associated with OS and possibilities of tumor recurrence of ICC patients. In multivariable analysis, the OPN level was defined to be an independent prognostic factor for both OS and tumor relapse (Supplementary Table 2). To further evaluate the association of OPN with ICC metastasis, we analyzed OPN levels in the HiBEpiC cells and ICC cells with various metastatic potentials^18,19^. OPN levels of five established ICC cell lines were much higher than that of HiBEpiC cells, and the OPN level was significantly associated with the metastatic abilities of ICC cells (Fig. 1f). These suggest that OPN is closely correlated with dismal prognosis and metastatic potential of ICC.

### OPN promotes ICC cell growth, migration, and metastasis

**Fig. 2:**
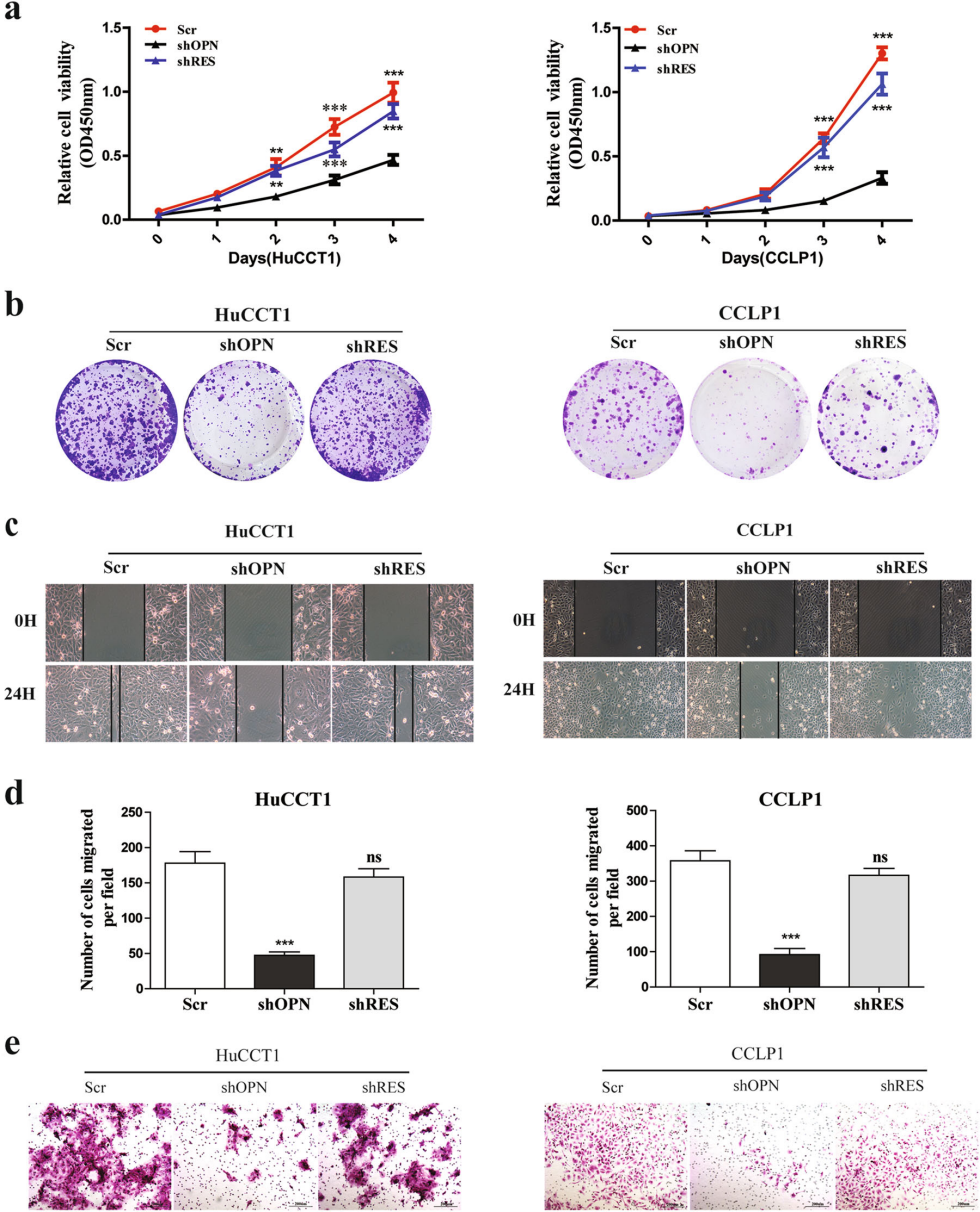
Knockdown of OPN inhibits ICC growth and metastasis in vitro. Knockdown of OPN significantly inhibited the **a** proliferation, **b** colony formation, **c** wound healing, **d** migration, and **e** invasion of HuCCT1 (left) and CCLP1 (right) cells, which were restored by shOPN-3-resistant OPN (shRES). Scr scrambled shRNA. All assays were performed in triplicate wells and repeated three times for each cell line, scale bar = 200 μm

To further explore the functional roles of OPN in ICC, OPN was knocked down in HuCCT1 and CCLP1 cells, which showed higher invasive capabilities and OPN expression. Three OPN-specific shRNAs were designed to silence the endogenous OPN expression in ICC cells and shOPN-3 which induced a 90% reduction of OPN expression was used for further studies (Supplementary Figure 1A and B). Then we reintroduced OPN in the OPN knocked down cell lines with a lentivirus expressing shOPN-3-resistant OPN (shRES) to exclude the possibility of offtarget effects and to check whether it could rescue the phenotypes caused by OPN down-regulation (Supplementary Figure 1C). Compared with the control cells, knockdown of OPN resulted in significant inhibition on the proliferation (Fig. 2a), colony formation (Fig. 2b and Supplementary Figure 1D), wound healing (Fig. 2c), migration (Fig. 2d and Supplementary Figure 1E), and invasion (Fig. 2e and Supplementary Figure 1F) of HuCCT1 and CCLP1 cells. Reintroducing OPN with shRES in HuCCT1-shOPN and CCLP1-shOPN cell lines could rescue the phenotypes induced by OPN knockdown. To further confirm these findings, Flag-OPN or empty vector was transfected into RBE, HCCC9810, and HuH-28 cell lines and found that exogenous expression of OPN remarkably enhanced the growth (Supplementary Figure 2A and B), migration (Supplementary Figure 2C), and invasion (Supplementary Figure 2D) of ICC cells compared with the control. These results indicate that OPN promotes ICC cell in vitro growth and metastasis.

**Fig. 3:**
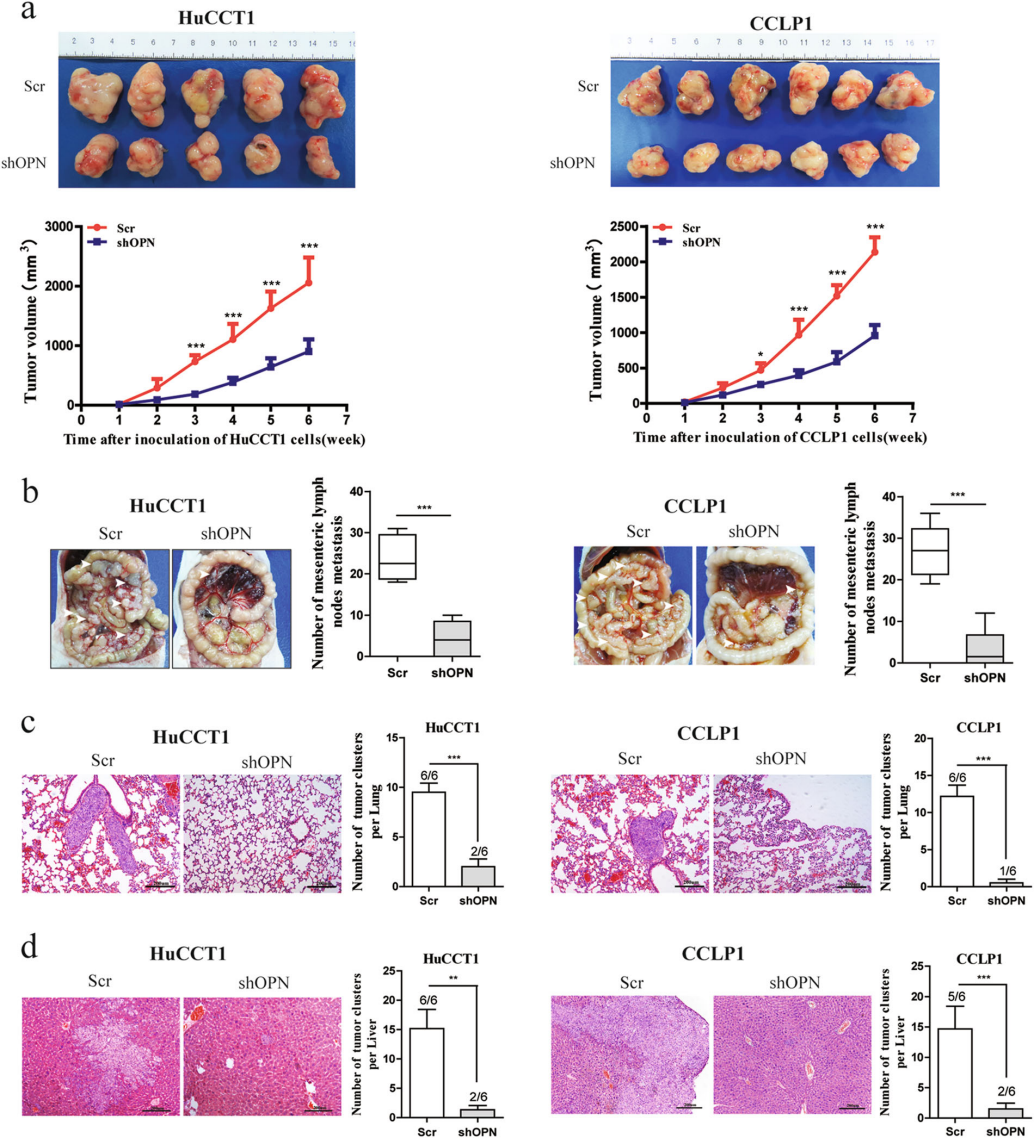
Knockdown of OPN reduces ICC growth and metastasis in vivo. **a** ICC cell lines with OPN knockdown and control were injected subcutaneously into nude mice (one mouse died 3 weeks later both in HuCCT1-shNC group and HuCCT1-shOPN group). The dynamic change of tumor volume in subcutaneous models was shown at 6 weeks after injection. **b** Knockdown of OPN decreased ICC metastasis in vivo. Stable OPN knockdown cell lines were injected into the intraperitoneal cavity of nude mice (*n* = 6), mice were sacrificed after 6 weeks, and the number of mesenteric lymph nodes metastasis per mouse was counted. (**c**,** d**) Down-regulation of OPN significantly inhibited lung (**c**) and liver (**d**) metastasis in ICC nude mice models. Scale bar = 200 μm

To evaluate the effect of OPN on ICC growth and metastasis in vivo, HuCCT1 and CCLP1 cell lines with OPN stable knockdown were subcutaneously implanted into the flank of nude mice and tumor size was monitored every week. As shown in Fig. 3a, the tumor volume decreased significantly in the OPN knockdown group compared to the control group. To further evaluate the effects of OPN on metastasis, HuCCT1 and CCLP1 cell lines with OPN knocked-down were injected into the intraperitoneal cavity of nude mice^18,20^, mice were sacrificed after 6 weeks, and the numbers of mesenteric lymph nodes, lung, and liver metastasis per mouse were counted. We found that suppressing OPN expression could remarkably reduce the metastases to mesenteric lymph nodes (Fig. 3b), lung (Fig. 3c), and liver (Fig. 3d) in mice models.

### OPN is demonstrated to interact with β-Catenin in ICC

**Fig. 4:**
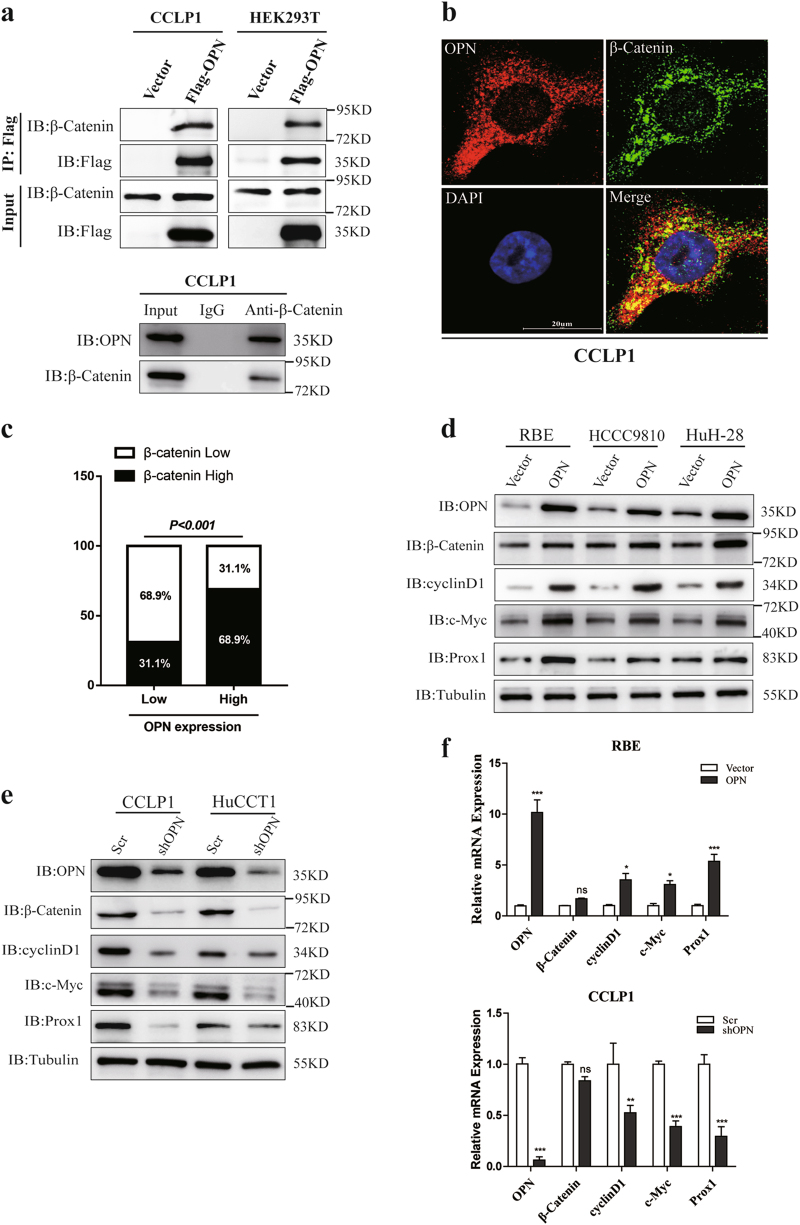
OPN interacts with β-Catenin in ICC. **a** Interaction between both exogenous and endogenous OPN and β-Catenin. CCLP1 and HEK293T cells were transfected with empty vector or Flag-OPN and subjected to Co-IP and IB with anti-Flag antibody. Interaction of endogenous OPN with β-Catenin was detected by IP using anti-β-Catenin antibody with IgG as control. **b** Co-localization of OPN (red) and β-Catenin (green) in CCLP1 cells by IF with anti-β-Catenin and anti-OPN antibody respectively. DAPI was used for nuclei staining. **c** OPN and β-Catenin expression were positively correlated in 180 ICC tumor tissues. **d**, **e** ICC cell lines were subjected to WB analysis. Overexpression of OPN in RBE, HCCC9810, and HuH-28 cells could obviously promote the protein level of β-Catenin, cyclin-D1, c-Myc, and Prox1 comparing to control (**d**). Knockdown of OPN in HuCCT1 and CCLP1 cells significantly inhibit the Wnt/β-Catenin pathway (**e**). **f** The qRT-PCR result of ICC cells with OPN overexpression (RBE) or knockdown (CCLP1). OPN modulated the mRNA expression of *C-MYC*, *CYCLIN-D1,* and *PROX1*, which are the target genes of β-Catenin. Scale bar = 20 μm

To reveal the underlying mechanisms of OPN in promoting ICC growth and metastasis, a combination of immunoaffinity purification and mass spectrometry was conducted to identify the interactors associated with OPN. CCLP1 cells transfected with Flag-OPN or empty vector were subjected to immunoprecipitation by anti-Flag mAb. Silver staining and MS was implemented to visualize and identify the coprecipitated proteins and found that β-Catenin, with two matching peptides, was a potential interactor of OPN (Supplementary Figure 3A). The association of both exogenous and endogenous OPN with β-Catenin was verified in HEK293T and CCLP1 cells by co-immunoprecipitation (co-IP) (Fig. 4a). Moreover, confocal microscopy demonstrated that OPN and β-Catenin were co-localized both in ICC cells (Fig. 4b and Supplementary Figure 3B) and patients (Supplementary Figure 3C and D). Then we analyzed the expression of OPN and β-Catenin in 180 ICC tissues by IHC and found that the expression level of β-Catenin was strongly related to OPN level in ICC tissues (*P*< 0.0001, Fig. 4c and Supplementary Figure 3E).

To further assess the relationship between OPN expression and the activation of the downstream of β-Catenin, we evaluated the expression of β-Catenin in ICC cell lines with OPN overexpression or knockdown. Up- or down-regulation of OPN lead to a corresponding increase or decrease of the protein level of β-Catenin, as well as the expression of target genes of Wnt signaling, such as *C-MYC*, *CYCLIN-D1,* and *PROX1* in ICC cell lines. However, no significant effect of OPN on β-Catenin mRNA level was observed (Fig. 4d–f and Supplementary Figure 4A and B).

Taken together, these results indicate that OPN interacts with β-Catenin to activate Wnt/β-Catenin pathway.

### OPN promotes ICC metastasis through regulating β-Catenin

**Fig. 5:**
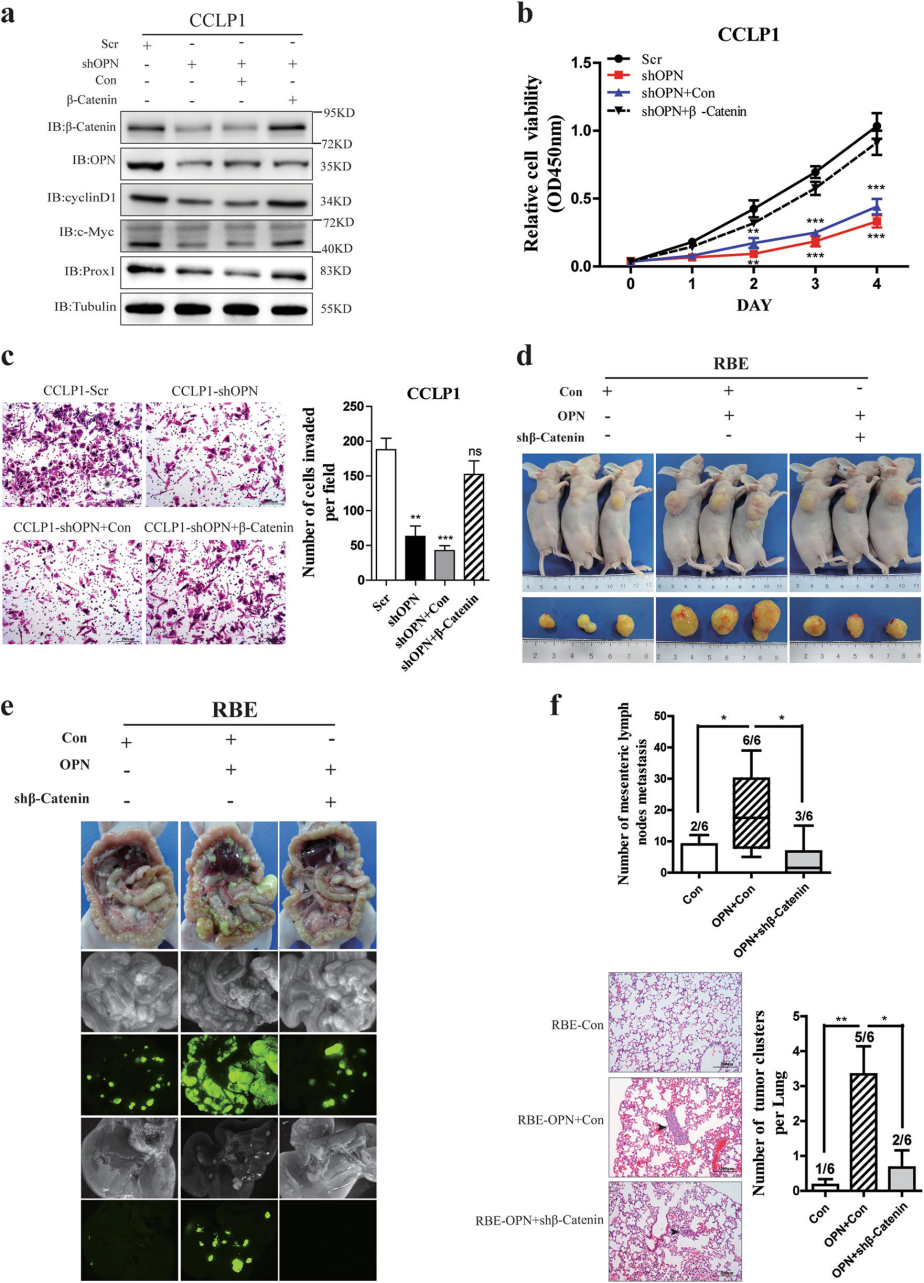
OPN promotes ICC progression via interacting with β-Catenin and activating Wnt signaling. **a** Down-regulation of OPN inhibited the Wnt/β-Catenin pathway, while β-Catenin overexpression could rescue the expression of cyclin-D1, c-Myc, and Prox1 caused by OPN knockdown. **b**, **c** Knockdown of OPN significantly suppressed the proliferation (**b**) and invasion abilities (**c**) of CCLP1 cells, which was counteracted by β-Catenin overexpression. **d** RBE cells (5 × 10^5^) stably expressing empty vector, Flag-OPN, or Flag-OPN plus shβ-Catenin were injected subcutaneously into nude mice. Tumor volumes of subcutaneous implantation models of ICC were shown. **e**, **f** OPN promotes ICC metastasis in vivo. Nude mice were implanted with RBE stable cell lines described as above into the intraperitoneal cavity; the mesenteric lymph and lung metastatic nodes were counted under a microscope

To explore whether OPN exerts its functions through β-Catenin, we investigated the effects of β-Catenin up-regulation on cancer development and progression of ICC cells with knockdown of OPN. Up-regulation of β-Catenin could rescue the decreased expression of the downstream genes of Wnt signaling pathway in ICC cells suppressed by knockdown of OPN (Fig. 5a), and enhanced in vitro cell proliferation (Fig. 5b), colony formation (Supplementary Figure 5A), migration (Supplementary Figure 5B), and invasion (Fig. 5c) of ICC cells inhibited by OPN knockdown.

To further confirm these finding, we established subcutaneous implantation nude mice models using RBE cells with OPN overexpression but β-Catenin knockdown, and found that up-regulation of OPN significantly enhanced tumor growth (Fig. 5d and Supplementary Figure 5C), metastases to the mesenteric lymph nodes (Fig. 5e,f), lung (Fig. 5f) and liver (Supplementary Figure 5D). However, β-Catenin knockdown reversed these enhancements, no significant difference was found in the tumor sizes and metastasis incidences between RBE cells with OPN/shβ-Catenin and the controls. These indicate that OPN promotes tumor growth and metastasis through regulating Wnt/β-Catenin pathway.

### OPN induces S675 phosphorylation and nucleus accumulation of β-Catenin in ICC

**Fig. 6:**
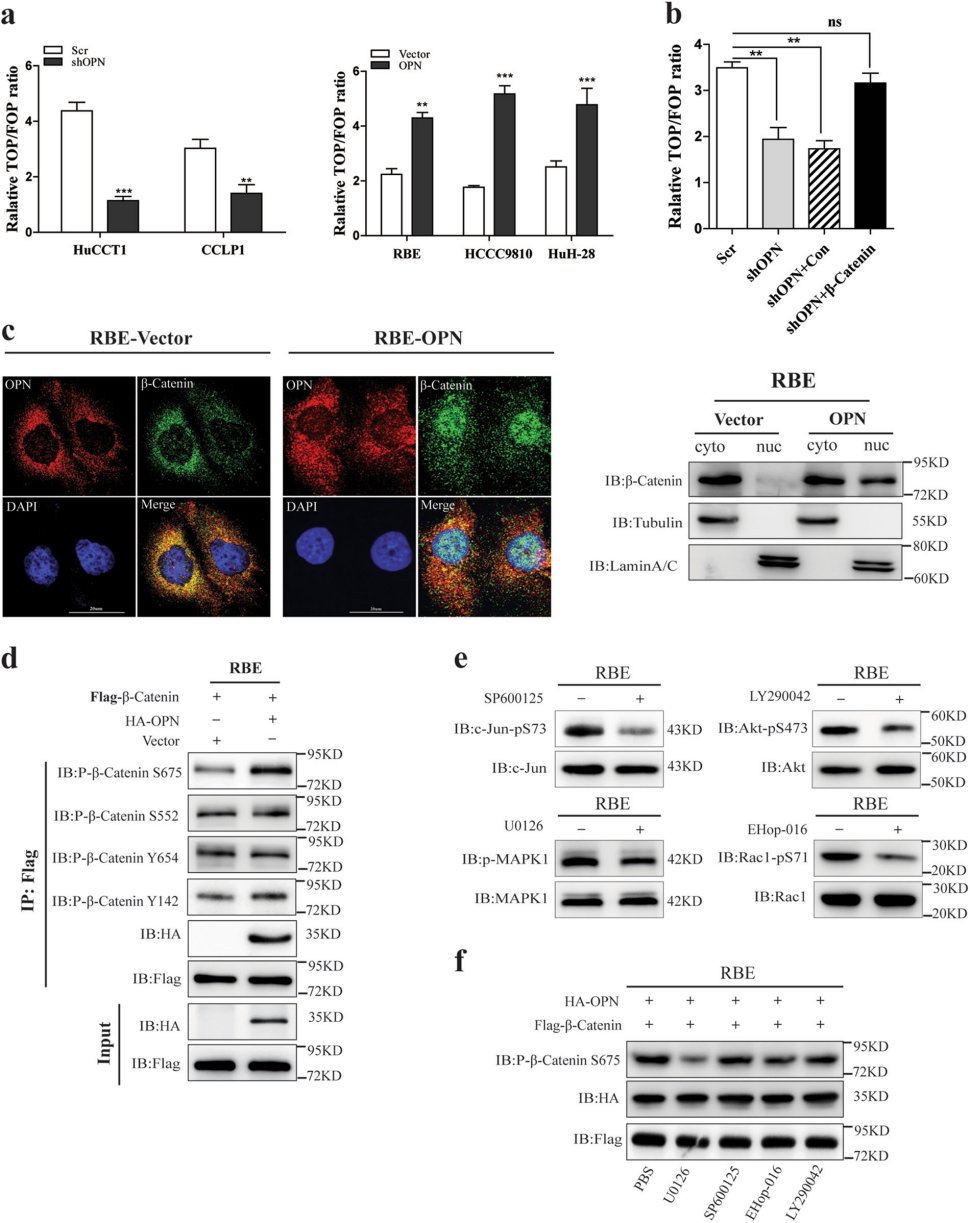
OPN promotes nuclear translocation of β-Catenin by phosphorylating it at Ser675 and activates Wnt signaling pathway. **a** The activity of the TOP/FOP reporter plasmid was analyzed by luciferase reporter assays in ICC cell lines with OPN up- or down-regulation. **b** β-Catenin could restore the activity of TOP/FOP reporter plasmid induced by OPN knockdown. **c** Immunofluorescence images of RBE cells with empty vector or OPN over-expressed (left). OPN, β-Catenin, and cell nuclei (DAPI) were exhibited as red, green, and blue, respectively. Cell lysates were subjected to the nuclear and cytosol fractionation, and β-Catenin expression was analyzed by western blot (right). **d** OPN was stably over-expressed in RBE cells and cell lysates were subjected to Co-IP; the phosphorylation of β-Catenin at S675, S552, Y654, and Y142 was analyzed by western blot. **e** The activity of kinases related to Wnt signaling activation was evaluated in RBE cells, which stably expressed empty vector or OPN and pretreated with U0126 (10 μM), EHop-016 (10 μM), *SP*600125 (20 μM), and LY290042 (20 μM) for 24 h. **f** Co-IP analysis of phosphorylated β-Catenin at Ser675 and OPN-β-Catenin complex was determined by western blot in RBE cells with Flag-β-Catenin and HA-OPN overexpression and pretreated with U0126, EHop-016, *SP*600125, and LY290042 for 24 h

Next, we performed TOP/FOP-FLASH reporter assay, which indicates the transcriptional activity of β-Catenin/TCF^21–23^, to further examine how OPN to modulate the Wnt/β-Catenin pathway. We found that knockdown of OPN failed to activate TOP/FOP-FLASH reporter expression in HuCCT1 and CCLP1 cells (Fig. 6a, left), while over-expressing OPN could significantly increase Wnt signaling activity in ICC cells (Fig. 6a, right).

Furthermore, knockdown of β-Catenin blocked the TOP/FOP reporter activity induced by OPN, which could be rescued by re-expression of β-Catenin (Fig. 6b). In addition, confocal images showed that OPN up-regulation induced a nucleus accumulation of β-Catenin (Fig. 6c, left). These results were further confirmed by cytosolic and nuclear fraction assays in RBE cells (Fig. 6c, right) and HCCC9810 cells (Supplementary Figure 5E). It is known that the phosphorylation of different sites of β-Catenin may affect its nuclear accumulation or protein stability and signaling activity^24–29^. Therefore, six of the potential phosphorylation sites which have been frequently reported to promote β-Catenin nuclear translocation and protein stability were subjected to IP-western analysis to identify the phosphorylation sites of β-Catenin mediated by OPN. Among six phosphorylation sites, only phosphorylation of Ser675 was regulated by OPN, but no obvious effect on the other five sites were observed (Fig. 6d and Supplementary Figure 5F). To investigate the downstream signaling involved OPN-mediated Ser675 phosphorylation of β-Catenin, we used various inhibitors to suppress the kinases related to Wnt signal activation including MEK-(MAP) kinases (U0126)^2^, c-Jun N-terminal kinase (JNK) (SP600125), PI3 kinase-AKT (LY294002), and Rac1 (EHop-016) (Fig. 6e). Western blot results showed that only MAPK inhibitor (U0126) completely blocked the Ser675 phosphorylation induced by OPN (Fig. 6f). These findings suggest that OPN induces Ser675 phosphorylation and nuclear accumulation of β-Catenin through MEK/MAPK. This is consistent with our previous report in HCC^13^.

### OPN recruits and activates MAPK1 to induce β-Catenin Ser675 phosphorylation and promotes ICC progression

**Fig. 7:**
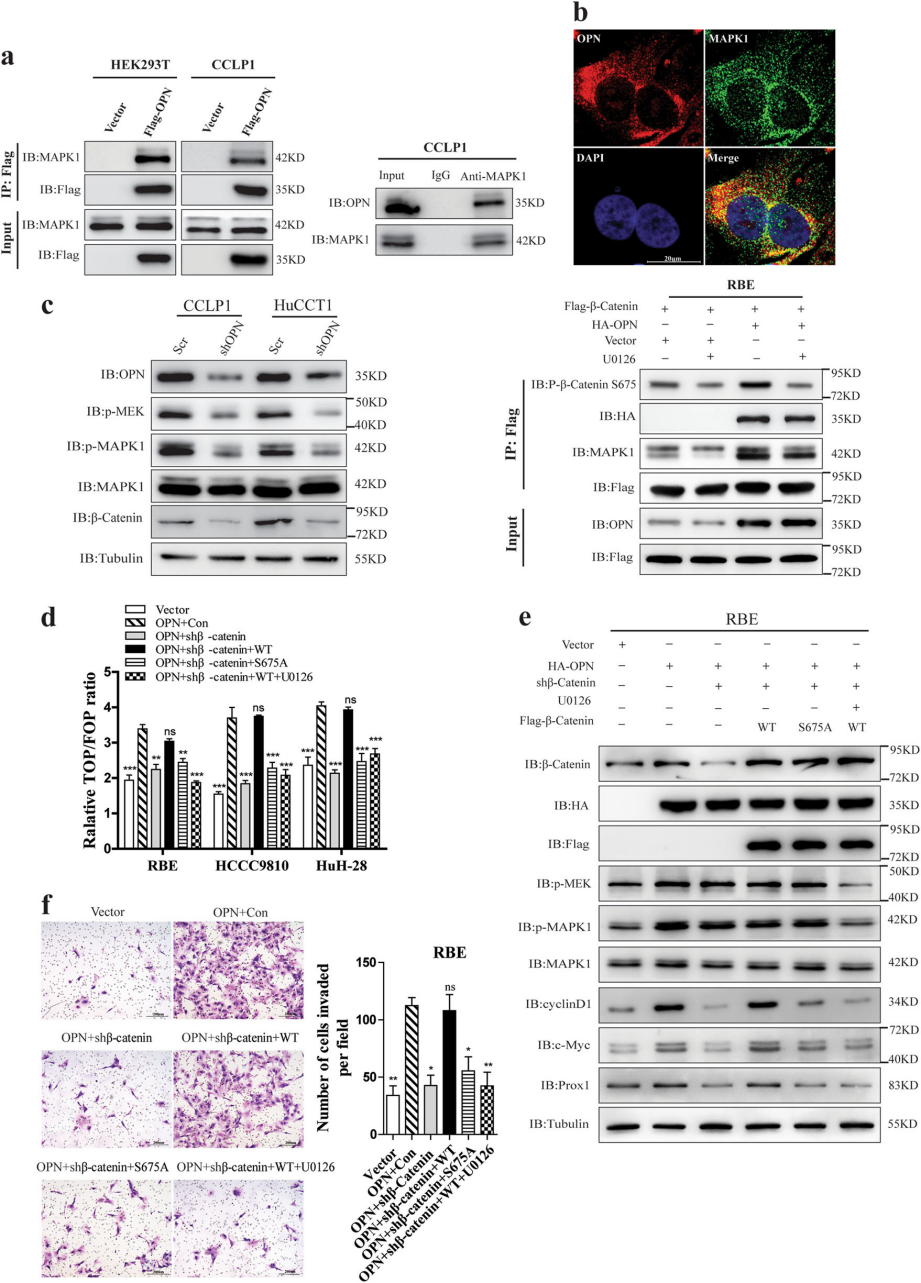
OPN recruited and activated MAPK1 to promote β-Catenin S675 phosphorylation and ICC metastasis. **a** Association of OPN with MAPK1 in HEK293T and CCLP1 cells. Interaction between exogenous OPN and MAPK1 in the left, CCLP1 cells and HEK293T cells was transfected with empty vector or Flag-OPN, total cell lysates was prepared for IP and IB using anti-FLAG or anti-MAPK1 antibodies. Endogenous MAPK1 in CCLP1 cells was determined by Co-IP with anti-MAPK1 monoclonal antibody, and rabbit IgG was used as nonspecific control. OPN was detected by anti-OPN antibody. **b** IF staining of OPN (red) and MAPK1 (green) in CCLP1 cells, Nuclei was stained with DAPI. **c** Knockdown of OPN in HuCCT1 and CLLP1 cells. IB examinations of both phosphorylated MEK (Ser217/221) and MAPK1 (Thr202/Tyr204) are shown in the left panel. RBE cells stably expressing Flag-β-Catenin were transfected with empty vector or HA-OPN and treated with 10 μM U0126 for 2 h, cell lysates were prepared, after that IP and IB were performed with anti-FLAG or specific antibodies indicated. The phosphorylation of β-Catenin S675 and OPN-MAPK1 complex was analyzed (right). **d** The TOP/FOP reporter plasmid was transfected and Luciferase reporter assays were performed in ICC cells. RBE, HCCC9810, and HuH-28 cells, with OPN or empty vector over-expressed and β-Catenin knockdown, were then rescued by wild-type β-Catenin (following U0126 treatment or not) or S675 mutants. **e** The activation of MEK/MAPK1 pathway and Wnt signaling pathway was determined with antibodies indicated. **f** Knockdown of β-Catenin could inhibit the invasion ability of CCLP1 cells induced by OPN, which could be restored by re-inducing β-Catenin-WT but not S675 mutants (S675A) or re-inducing β-Catenin-WT plus U0126 treatment. Scale bar = 20 μm

Since MAPK1 (ERK2) has been reported to play a crucial role in regulating the Wnt/β-Catenin signaling pathway^21^, we assumed that OPN could recruit and activate MAPK1 to regulate Wnt signaling. Co-IP demonstrated that both exogenous and endogenous OPN interacted with MAPK1 in CCLP1 and HEK293T cells (Fig. 7a). Confocal microscopy displayed a co-localization between endogenous OPN and MAPK1 in CCLP1 cells (Fig. 7b). Moreover, knockdown of OPN significantly decreased the protein levels of p-MEK, p-MAPK1, and β-Catenin (Fig. 7c left), OPN up-regulation distinctly activated the MEK/MAPK pathway and β-Catenin expression in RBE cells, and this activation of MEK/MAPK pathway was abrogated by MEK inhibitor U0126 (Supplementary Figure 6A).

Next, we detected whether OPN induced the Ser675 phosphorylation of β-Catenin through recruiting and activating MAPK1. Over-expressing OPN in RBE cells could remarkably induce β-Catenin Ser675 phosphorylation, which could be blocked by U0126 (Fig. 7c, right). Up-regulation of OPN and MAPK1 could remarkably increase β-Catenin Ser675 phosphorylation (Supplementary Figure 6B), while knockdown of MAPK1 and OPN almost eliminated the phosphorylation of β-Catenin at Ser675 (Supplementary Figure 6C). Both the western blot and IHC staining (Supplementary Figure 6D and E) in subcutaneous tumor tissues further confirmed that OPN down-regulation led to the suppression of MEK/MAPK1 pathway and Wnt signaling.

Down-regulation of β-Catenin blocked the TOP/FOP reporter activity mediated by OPN overexpression. However, there is no significant effect on Wnt/β-Catenin transcriptional activity in RBE cells when re-expression of β-Catenin shRES following with U0126 treatment or re-expression of β-Catenin S675A, which mimicked unphosphorylation of β-Catenin at S675 (Fig. 7d). OPN up-regulation activated the MEK/MAPK1 pathway and Wnt signaling, but inhibition of β-Catenin expression decreased the expression of c-Myc, cyclin-D1, and Prox1. Re-expression of β-Catenin shRES, rather than β-Catenin S675A, could restore the activation of Wnt signaling. Nonetheless, treatment of U0126 which was the inhibitor for MEK/MAPK1 could also inhibit the activity of Wnt signaling caused by OPN overexpression or rescuing effect by re-expression of β-Catenin shRES (Fig. 7e).

As expected, re-introduction of β-Catenin could restore in vitro colony formation (Supplementary Figure 7A) and cell invasion (Fig. 7f and Supplementary Figure 7B) of ICC cells. However, inhibition of β-Catenin Ser675 phosphorylation or MAPK1 activation significantly inhibited in vitro colony formation (Supplementary Figure 7A) and cell invasion (Fig. 7f and Supplementary Figure 7B) induced by OPN. Taken together, these results demonstrate that OPN promotes the growth and metastasis of ICC cells by recruiting and activating MAPK1 to induce β-Catenin Ser675 phosphorylation and activation of the Wnt/β-Catenin pathway.

### Combination of OPN and β-Catenin expression has a better performance in prognostic prediction for ICC

**Fig. 8:**
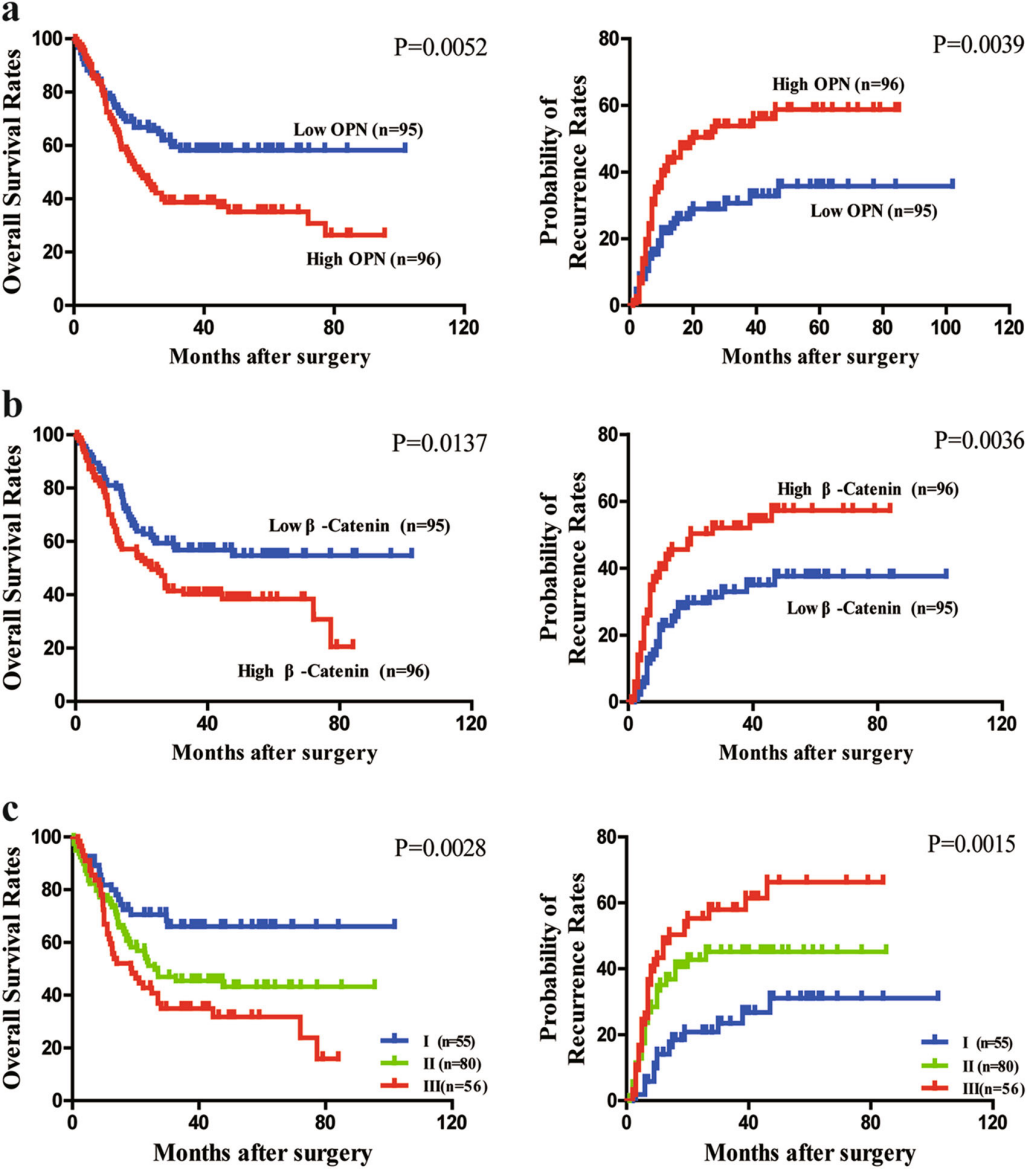
Prognostic value of OPN and β-Catenin for ICC patients. **a** ICC patients with high OPN expression have a shorter OS and higher probability of recurrence compared with low OPN expression patients. **b** β-Catenin expression levels were correlated with the OS and probabilities of tumor relapse of ICC patients. **c** Significant differences in OS and probabilities of tumor relapse among three different subgroups based on the combination of OPN and β-Catenin, i.e., group I, low OPN/low β-Catenin; group II, high OPN/low β-Catenin or low OPN/high β-Catenin; and group III, high OPN/high β-Catenin. Different subgroups were analyzed according to the cutoff values of OPN and β-Catenin levels, which were defined as the median value. The patients with low OPN/low β-Catenin had the best clinical outcomes, while the patients in group III showed the shortest OS and highest possibility of tumor relapse

To excavate the prognostic significance of OPN and β-Catenin levels for ICC patients, IHC staining was performed using ICC tissue microarrays (TMAs) containing 180 patients from an independent cohort. Patients with high OPN levels exhibited higher preoperative GGT level, advanced pTNM stage, and more regional lymph node metastasis (Supplementary Table 3) compared with those low OPN ones. There were no significant correlation between β-Catenin level and most of the clinicopathological features, except tumor thrombus. Kaplan–Meier analysis showed that patients with high expression of OPN or β-Catenin could gain poor prognosis including shorter OS and lower possibility of postoperative relapse (Fig. 8a, b). Univariate analysis indicated tumor differentiation, pTNM stage, OPN, and β-Catenin expression were closely associated with OS and possibility of recurrence of ICC patients (Supplementary Table 4). Furthermore, multivariate analysis confirmed that CA19-9 level, tumor differentiation, pTNM stage, OPN, and β-Catenin were independent prognostic factors for both OS and possibility of recurrence for ICC patients (Supplementary Table 4).

Strikingly, ICC patients with high levels of OPN and β-Catenin had the most dismal prognosis (Fig. 8c). Univariate and multivariate analysis also demonstrated OPN, β-Catenin, and their combination were an independent prognostic role for OS and possibility of recurrence (Supplementary Table 4). The receiver operating characteristic curve (ROC) revealed this combined predictive algorithm appeared to have a better predictive capacity compared with either alone group (Supplementary Figure 8A and B).

## Discussion

ICC is a devastating malignancy, with an extremely poor 5-year survival and high recurrence rate after resection^5,22^. The molecular pathogenesis of ICC involved multiple signaling pathway deregulations and aberrant activation of Ras–MAPK^30^, Wnt signaling^7,19^, JAK–STAT3^18,31^ and so on. Nevertheless, an in-depth exploration of the pivotal factors driving tumorigenicity and metastasis of ICC is urgently needed to identify new biomarkers for ICC prediction and targeted therapy.

Previous studies found that OPN modulates cell migration, EMT, ECM-invasion and stemness maintaining of tumor cell via binding integrins and CD44 or activating NF-Kb, MEK/MAPK, PI3K/Akt, and FAK pathways^9,11,13,14,17^. In ICC, OPN was reported to be upregulated both in tumor tissues and stroma^16,17^. In the present study, we found that OPN level was significantly increase both in the plasma and tumor tissues, which was associated with regional lymph node metastasis and prognosis. These data suggest that OPN might play a pivotal role in the malignant progression of ICC.

Both the in vivo and in vitro functional studies demonstrated that OPN knockdown can significantly inhibit the proliferation, colony formation, migration, and invasion ability in vitro as well as the tumor growth and lymph nodes metastasis in nude mice. In the mechanism study, we disclosed that OPN interacted with β-Catenin and activated the Wnt signaling pathway, as well as the expression of downstream target gene, such as *C-MYC*, *CYCLIN-D1*, and *PROX1*, through which it promoted the growth and metastasis of ICC.

Aberrant activation of the Wnt signaling pathway has been observed in ICC progression because of the nucleus accumulation of β-Catenin^19,32,33^. β-Catenin is the major effector of the Wnt signaling pathway. In this study, we found that OPN knockdown inhibited the mRNA and protein level of cyclin-D1, c-Myc, and Prox1, which were the target genes of the Wnt/β-catenin signaling pathway, through down-regulation of the protein level not the mRNA level of β-catenin. Knockdown of β-catenin reversed the promotion of tumor growth and mesenteric lymph nodes induced by OPN overexpression in nude mice. Even more interesting was that the expression level of OPN was significant correlation with the β-Catenin level in ICC samples and the combination of OPN and β-Catenin levels was an independent prognostic indicator for OS and recurrence of ICC patients.

β-Catenin is frequently rapidly targeted by the complex made of APC (adenomatous polyposis coli protein), GS3Kβ (glycogen synthase kinase-3b), axin, and casein kinase 1a for proteasome degradation after phosphorylation. Recently, different studies have proved that the phosphorylation of specific sites could promote the stability and nucleus translocation of β-Catenin. The JNK2-mediated Ser191 and Ser605 phosphorylation^27^, Rac1/PAK1-mediated S675 phosphorylation^29^, Akt-mediated Ser552 phosphorylation^24,34^, c-Met kinase-mediated Y142 phosphorylation^25,26,35^, and HGF-mediated Y654 phosphorylation^28^ could improve the stability and nucleus accumulation of β-Catenin. We found that OPN overexpression significantly increased the Ser675 phosphorylation of β-Catenin, which further promoted the nucleus accumulation of β-Catenin and activation of the transcription of Wnt target genes.

Our previous study found that OPN promoted HCC growth and metastasis^13^. Here, we revealed that OPN could recruit and activate MAPK1 to induce β-Catenin Ser675 phosphorylation and promote ICC progression. Inhibition of the MEK/MAPK1 pathway by U0126, an inhibitor for MEK/MAPK1 signaling, or overexpression of β-Catenin S675A (serine to alanine) could block the activation of Wnt signaling and promotion of growth and metastasis of ICC cells induced by OPN.

In conclusion, our study revealed OPN as a novel promoter of ICC malignant progression. Moreover, OPN selectively interacted with β-Catenin and mediated its S675 phosphorylation through recruiting and activating MAPK1 resulting in activation of the Wnt signaling (Supplementary Figure 9). These data suggest that OPN plays critical roles in tumor growth and metastasis and may become a promising prognostic indicator and potential therapeutic target for ICC metastasis.

## Materials and methods

### Patients, specimens, and follow-up

Total 387 patients were enrolled in this study. Paired tumor and adjacent non-tumor liver tissues were collected from patients who underwent surgical resection for ICC in authors’ institutions with a written informed consent. The tissue specimens were immediately frozen in liquid nitrogen or fixed in 10% formalin and paraffin embedded. No patient had received any anti-tumor treatment preoperatively. This study was approved by the Ethics Committee of Fudan University.

In all 387 patients, 31 individuals and another 29 healthy donors were extracted their plasma for analysis; paired tissues from 54 patients were collected for detecting the OPN level. The remaining 302 patients were divided into two independent cohorts for prognosis analysis. Cohort 1 included 122 patients with ICCs and cohort 2 included 180 cases of ICCs.

### TMA and IHC

TMA blocks were constructed with tumors and corresponding adjacent non-tumor tissues from the ICC patients, IHC staining procedure as described in the supplementary material. Image-Pro Plus v6.0 software (Media Cybernetics Inc., Bethesda, MD, USA) was used to assess the immunostaining according to the mean optical density, as previously described^36^. The cutoff of OPN or β-Catenin was defined as the mean of the values.

### Immunoprecipitation (IP), silver staining, and mass spectrometry

IP and mass spectrometry were performed as described previously^37^; details are described in the supplementary material.

### Co-IP and western blot

Co-IP and western blot were performed as described previously^37^. All antibodies used are listed in supplementary material, Table S6.

### TOP-Flash assay

ICC cells were transiently transfected with the TOP-flash or FOP-flash reporter plasmid (Upstate, Lake Placid, NY) by using FuGene HD reagent (Roche, Indianapolis, IN). In order to normalize transfection efficiency, cells were cotransfected with 0.2 mg Renilla reniformis luciferase driven under the TK promoter (pRL-TK; Promega, Madison, WI), which was usually used as an internal control reporter. Luciferase assay was performed by using the Dual Luciferase Assay System kit 36 h after transfection according to the manufacturer’s protocols (Promega). Relative luciferase activity was reported as fold induction after normalization for transfection efficiency.

### In vitro and in vivo assays

The in vivo mice model was established as described previously^18,20,37^. Details about in vitro and in vivo assays can be found in the supplementary material.

### Statistical analysis

Statistical analysis was performed using SPSS version 13.0 and Graphpad 6.0. Qualitative data are presented as number (percent), and quantitative values were shown as mean ± SD. The association between qualitative variables was analyzed using the Chi-square test or Fisher’s exact test, while quantitative values using Student's *t*-test or Mann–Whitney test, when appropriate. The impact of prognostic factors on OS and recurrence of ICC patients was assessed using Cox regression and Kaplan–Meier analysis. ROC analysis was used to find the predictive value of each indicator. Two-tailed *P* < 0.05 was regarded as of statistical significance marked with *, similarly *P* < 0.01 with ** and *P *< 0.001 with ***.

Detailed methods for immunohistochemistry, ELISAs, RNA isolation and real-time qPCR, cell culture and plasmids, immunoprecipitation, silver staining and mass spectrometry, nuclear and cytosol fractionation assay, immunofluorescence, cell proliferation assay, colony formation assay, wound-healing assay, migration and invasion assay, in vivo tumor growth, and metastasis assays are described in the supplementary material, supplementary materials and methods.

## Electronic supplementary material

Supplementary material

Supplementary Figure1

Supplementary Figure2

Supplementary Figure3

Supplementary Figure4

Supplementary Figure5

Supplementary Figure6

Supplementary Figure7

Supplementary Figure8

Supplementary Figure 9
