# Supplementary material for: Osteopontin promotes metastasis of intrahepatic cholangiocarcinoma through recruiting MAPK1 and mediating Ser675 phosphorylation of β-Catenin
Source: Cell Death Dis. 2018 Feb 7;9(2):179. doi: 10.1038/s41419-017-0226-x (PMC5833342; doi:10.1038/s41419-017-0226-x)
Supplement: Supplementary file 1 — Supplementary material [file 41419_2017_226_MOESM1_ESM.docx]

**Osteopontin** promotes metastasis of intrahepatic cholangiocarcinoma through recruiting MAPK1 **and mediating** Ser675 phosphorylation of β-catenin

Yan Zheng^#^, Chuang Zhou^#^, Xin-Xin Yu^#^, Chao Wu, Hu-Liang Jia, Xiao-Mei Gao, Ji-Meng Yang, Chao-Qun Wang, Qin Luo, Ying Zhu, Yu Zhang, Jin-Wang Wei, Yuan-Yuan Sheng, Qiong-Zhu Dong*, Lun-Xiu Qin*

**Table of Contents**

- Abbreviations
- Author contributions statement
- Supplementary Materials and Methods
- Supplementary tables
- Supplementary figure legends

**Abbreviations:**

ICC, intrahepatic cholangiocarcinoma; OPN, osteopontin; OS, overall survival; GGT, γ-glutamyl transpeptadase; IHC, immunohistochemistry; TMA, tissue microarray; ELISAs, enzyme-linked immunosorbent assays; IB, immunoblotting; qRT-PCR, quantitative real time polymerase chain reaction; Co-IP, coimmunoprecipitation; MAPK1, mitogen-activated protein kinase 1; MEK, MAP kinse; JNK, c-Jun N-terminal kinases; Rac1,ras-related C3 botulinum toxin substrate 1; WT, Wild-type.

**Author contributions statement**

Conception and design: YZ, CZ; Development of methodology: YZ, XXY; Provision of animals and facilities: YZ, XXY; Enrollment and management of patients information: CW, XMG; Statistical analysis: CW, HLJ, JMY, CQW; Writing, review and revision of the manuscript: YZ; Reporting or organizing data: QL, YZ; Administrative, technical and material support: YZ, JWW and YYS; Study supervision: QZD and LXQ.

**Supplementary Materials and Methods**

**Immunohistochemistry (IHC)**

Immunohistochemical staining was performed on TMA slides by two steps. After deparaffinage and rehydration, the tissue slides were treated with 0.01mol/L sodium citrate (pH 6.0) in a microwave oven for 10 minutes for antigen retrieval. Then, sections were incubated with the monoclonal anti-OPN or anti-β-Catenin antibodies at 4℃ overnight and subsequently developed using ChemMate DAKO EnVision Detection Kit, Peroxidase/DAB, Rabbit/Mouse (Dako Cytomation, Glostrup, Denmark). Later, slides were counterstained with hematoxylin. All procedures were conducted consistently only with omitting primary antibody in negative controls.

**Enzyme-linked Immunosorbent Assays (ELISAs)**

The blood samples were obtained before operation and separated by centrifugation (10,000 g for 10 min) at 4°C, and then stored at -80°C until analysis. Double-blinded quantitative detection of plasma OPN was performed using the Human OPN ELISA kit (R&D Systems, USA), according to the manufacturer’s instructions.

**RNA Isolation and Real Time Quantitative Reverse Transcription PCR (Real Time qRT-PCR)**

RNA isolation and cDNA obtaining were performed as described previously[[1](#_ENREF_1)]. For qRT-PCR analysis, cDNA were amplified using SYBR Green Realtime PCR Master Mix (Takara, Japan). qRT-PCR reactions were performed in triplicates as the following conditions: 95 °C/20 s, 40 cycles of 95 °C/60 s and 60 °C/20 s on using the ABI PRISM 7900 Sequence Detection System (Applied Biosystems, Foster City, CA, USA) and repeated at least three times. Relative mRNA levels were analyzed by the -ΔΔCt method using β-actin as the endogenous control and presented as 2^^-ΔΔCt^. Primer sequences are as follow: Actin-F/R:CATGTACGTTGCTATCCAGGC/CTCCT

TAATGTCACGCACGAT,OPN-F/R:CTCCATTGACTCGAACGACTC/CAGGTCTGCGAAACTTCTTAGAT,β-catenin-F/R:AAAGCGGCTGTTAGTCACTGG/CGAGTCATTGCATACTGTCCAT,CyclinD1-F/R:GCTGCGAAGTGGAAACCATC/CCTCCTTCTGCACACATTTGAA,c-Myc-F/R:GTCAAGAGGCGAACACACAAC/TTGGACGGACAGGATGTATGC,PROX1-F/R:AAAGTCAAATGTACTCCGCAAGC/CTGGGAAATTATGGTTGCTCCT.

**Cell Culture and Plasmids**

Five human ICC cell lines (RBE/HCCC9810/HuH28/HuCCT1/CCLP1), one intrahepatic biliary epithelial cell line (HiBEpiC) and HEK293T cell were used in this study. ICC cell lines (HuH28/HuCCT1/CCLP1) and HiBEpiC cell were obtained from professor Kun-Liang Guan, Yue Xiong and Xiang-Huo He in Institutes of Biomedical Sciences, Fudan University. RBE, HCCC9810 and HEK293T cell were obtained from the the Cell Bank of Shanghai Institutes of Biological Sciences, Chinese Academy of Sciences. RBE, HCCC9810 and HiBEpiC were cultured in Dulbecco’s modified Eagle’s medium (Hyclone) and HuCCT1/CCLP1 were cultured in 1640 medium (Hyclone) supplemented with 10% fetal bovine serum (Gibco) with a 5% CO2 concentration at 37℃. In addition, HuH28 was cultured in MEMα medium (Gibco) supplemented with 15% fetal bovine serum (Gibco).

The lentivirus-mediated RNA interference of OPN, β-catenin and MAPK1 were cloned in pLKO.1 TRC (Addgene plasmid 10879) respectively. A scrambled siRNA precursor (Scr) was used as a negative control. The shRNA sequences for target genes in this paper were purchased from Sigma-Aldrich. The sequences of shRNAs targeting for OPN are as follows: shOPN-1:5'-CTTTACAACAAATACCCAGAT-3';

shOPN-2:5'-CGAGGAGTTGAATGGTGCATA-3';shOPN-3:5'-CCACAAGCAGTCCAGATTATA-3';The sequences of shRNA targeting for β-catenin are as follows: shβ-catenin-1: 5'-ATCTGTCTGCTCTAGTAATAA-3';shβ-catenin-2:5'-TTGTTATCA

GAGGACTAAATA-3';shβ-catenin-3:5'-TCTAACCTCACTTGCAATAAT-3'; The sequences of shRNAs targeting for MAPK1 are as follows: shMAPK1-1: 5'-CCCATATCTGGAGCAGTATTA-3'; shMAPK1-2: 5'-TATCCATTCAGCTAACGT

TCT-3'; shMAPK1-3:5'-CAAAGTTCGAGTAGCTATCAA-3'. Lentiviral overexpression vectors for OPN, β-catenin and MAPK1 were constructed by cloning c-terminal FLAG tagged target genes into pCDH-CMV-MCS-EF1-Puro (CD510B-1), which was obtained from system biosciences (USA). ShRNA resistant plamids for OPN and β-catenin were made by implementing site-directed mutations to introduce five silent mutations against OPN or β-catenin shRNA, primer sequences were as follow:OPN-F:5'-TGCTGAAACCCACAGTCATAAGCAATCTAGGTTATATAAGCGGAAAGC-3',OPN-R:5'-GCTTTCCGCTTATATAACCTAGATTGCTTATGACTGTGGGTTTCAGCA-3';β-catenin-F:5'-TTCACAACCGAATTGCTACCAAAGGACAAAGTACCATTCCATTGT-3',β-catenin-R:5'-ACAATGGAATGGTACTTTGTCCTTTGGTAGCAATTCGGTTGTGAA-3'. Plamid of β-catenin S675A, S191A, S605A was constructed by performing site-directed mutations to shRES for β-catenin plamid, primer sequences were as follow: S675A-F:5'- TACAAGAAACGGCTTGCAGTTG

AGCTGACCAGC-3',S675A-R:5'-GCTGGTCAGCTCAACTGCAAGCCGTTTCTTGTA-3';S191A-F:5'-CACGCTATCATGCGTGCTCCTCAGATGGTGTCT-3', S191A-R:5'- AGACACCATCTGAGGAGCACGCATGATAGCGTG -3';S605A-F:5'- GTGCAGCTGCTTTATGCTCCCATTGAAAACATC-3',S605A-R:5'-GATGTTTTCAATGGGAGCATAAAGCAGCTGCAC-3'.

**Immunoprecipitation(IP), Sliver Staining and Mass Spectrometry**

At least 1x10^8^ Flag-OPN or empty vector CCLP1 stable cell lines were lysed in RIPA buffer (10mM Tris-HCl, 150mM NaCl, 1% NP40, 5mM EDTA) at 4°C for 1 hour. Lysates were centrifuged at 12000 g, 4°C for 15 min. Supernatants were loaded to M2 anti-Flag mAb agarose beads (ANTI-FLAG® M2 Affinity Gel, Sigma). Then the beads were washed with RIPA buffer and the protein complex was eluted with FLAG peptides (Sigma) according to the manufacturer’s protocol. Eluates were subjected to SDS-PAGE followed by silver staining. The bands specific for the Flag-OPN overexpression were extracted and subjected to mass spectrometry analysis using ABI 4700 Protein Analyzer (TOF, ABI). Sliver staining was performed according to the manufacturer’s protocol (PROT-SIL2, sigma).

**Nuclear and Cytosol Fractionation Assay**

ICC cells were harvested and lysised in nori buffer containing protease inhibitor (pH7.0 20mM Hepes, 10mM KCl, 2mM MgCl2 and 0.5% NP-40). Cell lysates were centrifuged at 4000rpm, 4°C for 5min after incubated on ice for 10min and homogenized. Supernatants were centrifuged at 13000 rpm, 4°C for 20min, and then the supernatant could be saved as non-nuclear fraction.

Then the nuclear pellet should be washed with 1ml nori buffer for 3 times and resuspended with NETN buffer(150mM NaCl, 1mM EDTA, 20mM Tris pH8.0, 0.5% NP-40). The sample was then subjected to sonicate for 10sec, 2 times and centrifugation at 13000 rpm, 4°C for 30min. The supernatant could be saved as nuclear fraction.

**Immunofluorescence and Co-localization Assays**

ICC cells were plated into 6-well plate containing two cover glasses of 12 mm in diameter overnight and then were fixed in 4% paraformaldehyde for 15 min. After triple washing in PBS for 5 min, the cells were incubated in 0.5% Triton X-100 for 10 min at room temperature, and then incubated in 1% BSA for 1 hour followed by incubation with primary antibody at 4°C overnight. Cells were washed in PBS for 3 times followed by incubation with secondary Alexa488 or 594-conjugated anti-mouse or rabbit second antibody (Invitrogen, Carlsbad, USA) in 1% BSA at room temperature for 1 hour. After washing as described above, samples were counterstained with DAPI 100μg/ml in PBS for 5 min. Finally, the slides were sealed with a coverslip mounted in antifade reagent (Invitrogen, USA). Images were captured using confocal fluorescent microscope (Nikon, Tokyo, Japan).

**Cell Proliferation Assay**

Cell proliferation was measured by using Cell Counting Kit-8 (Dojindo, Kumamoto, Japan). ICC cell lines were seeded into 96-well plates at a density of 1×10^3^ cells per well (n=8 for each time point) in a final volume of 100 μl medium containing 10% FBS. Then cells were incubated at 37 °C in 5% CO2 for 24, 48, 72, and 96 hours and the medium was replaced with 10 μl CCK-8 solution and 90 μl of fresh medium each well. The absorbance at 450 nm was measured after incubation for 1 hours at 37°C in 5% CO2. Each experiment was repeated for three times on each condition.

**Colony formation assay**

ICC stable cell lines were seeded in six-well plates at a density of 8 × 10^2^ cells/well. Cells were incubated at 37 °C in 5% CO2 for about 10–14 days, then cells were fixed in 4% formalin for 15 minutes and stained with Giemsa solution (AppliChem, Darmstadt, Germany) after washed twice with PBS. After that cells were allowed to air dry at room temperature. The number of colonies was counted. Each experiment was performed for three times.

**Wound-Healing Assay**

ICC cell lines were cultured in monolayers overnight and wounded with a pipette tip. Then images were taken at 0 hours and 24 hours with light microscope. Each experiment was performed for three times.

**Migration and Invasion Assay**

To assay the ICC cell lines’ ability of migration and invasion, 24-well transwell chambers, with upper and lower culture com­partments separated by polycarbonate mem­branes with 8 μm pores (BD Pharmingen). The bottom chamber was filled with 10% FBS supplemented DMEM. 3×10^4^ cells without matrigel was prepared for migration, and 4×10^4^ cells with prepared for invasion 1 hour after seeding 100ul matrigel in the upper chamber. Then cells suspended in serum-free medium were then seeded into the upper chamber and maintained in normal cell culture condition mentioned above. Cells that migrated or invaded to the under­side of the membrane were stained with crystal violet, calculated with light microscope.

***In Vivo* Tumor Growth and Metastasis Assays**

The *in vivo* experimental protocols were approved by the Animal Ethics Committee of Shanghai Medical College, Fudan University. Five-week-old male nude mice (BALB/c) were housed and randomly divided into indicated groups (6 mice/group) before inoculation, and double-blinded evaluation was performed when measuring tumor weight, volume, and number of metastatic nodules. Stable ICC cell lines (HuCCT1-shNC, shOPN and CCLP1-shNC, shOPN; RBE-Vector, RBE-OPN, RBE-OPN+Siβ-Catenin) were subcutaneously implanted into nude mice (5×10^6^ cell/mouse) to establish the subcutaneous implantation model. The stable ICC cell lines were injected into the intraperitoneal cavity of nude mice (2.5*10^6^cells/mouse in 200ul PBS) to establish metastatic model. All mice were sacrificed 4 or 6 weeks later and their tumor, liver, lung and mesenteric lymph nodes metastasis were removed and analyzed. The whole liver, lung and tumor tissues were fixed with paraformaldehyde (4%) before dehydration and embedding in paraffin. Paraffin sections were stained with H&E according to standard protocols and mesenteric lymph nodes, lungs and liver metastatic nodules were counted under microscope.

**Supplementary Tables**

**Table 1. Comparison of clinicopathologic profiles between low and high OPN expression in ICC patients from Cohort 1 (n=122)**

| Variables | OPN Expression | | | | |
| --- | --- | --- | --- | --- | --- |
|  | Low(N=61) | | High(N=61) | | P |
|  | No. of patients | % | No. of patients | % |  |
| Gender* |  |  |  |  |  |
| Female | 24 | 39.3% | 22 | 36.1% | 0.709 |
| Male | 37 | 60.7% | 39 | 63.9% |  |
| Age* |  |  |  |  |  |
| ≤50 | 10 | 16.4% | 13 | 21.3% | 0.487 |
| ＞50 | 51 | 83.6% | 48 | 78.7% |  |
| HBsAg* |  |  |  |  |  |
| Negative | 37 | 60.7% | 46 | 75.4% | 0.120 |
| Positive | 24 | 39.3% | 15 | 24.6% |  |
| HBcAb* |  |  |  |  |  |
| Negative | 19 | 31.1% | 20 | 32.8% | 0.846 |
| Positive | 42 | 68.9% | 41 | 67.2% |  |
| Cirrhosis* |  |  |  |  |  |
| No | 40 | 65.6% | 44 | 72.1% | 0.434 |
| Yes | 21 | 34.4% | 17 | 27.9% |  |
| ALT(U/L)* |  |  |  |  |  |
| ≤75 | 45 | 73.8% | 51 | 83.6% | 0.185 |
| ＞75 | 16 | 26.2% | 10 | 16.4% |  |
| Tbil (μmol/dl) * | |  |  |  |  |
| ≤17.1 | 40 | 65.6% | 46 | 75.4% | 0.234 |
| ＞17.1 | 21 | 34.4% | 15 | 24.6% |  |
| GGT (U/L) * | |  |  |  |  |
| ≤60 | 36 | 59.0% | 22 | 36.1% | **0.011** |
| ＞60 | 25 | 41.0% | 39 | 63.9% |  |
| CA19-9(U/ml) * |  |  |  |  |  |
| ≤37 | 24 | 39.3% | 28 | 45.9% | 0.464 |
| ＞37 | 37 | 60.7% | 33 | 54.1% |  |
| Tumor size(cm) * | |  |  |  |  |
| ≤5 | 29 | 47.5% | 26 | 42.6% | 0.585 |
| ＞5 | 32 | 52.5% | 35 | 57.4% |  |
| Tumor number* |  |  |  |  |  |
| Single | 58 | 95.1% | 55 | 90.2% | 0.491 |
| Multiple | 3 | 4.9% | 6 | 9.8% |  |
| Tumor capsule* |  |  |  |  |  |
| None | 51 | 83.6% | 57 | 93.4% | 0.154 |
| Complete | 10 | 16.4% | 4 | 6.6% |  |
| Tumor thrombus* | |  |  |  |  |
| No | 51 | 83.6% | 49 | 80.3% | 0.638 |
| Yes | 10 | 16.4% | 12 | 19.7% |  |
| Tumor differentiation* | |  |  |  |  |
| I+II | 38 | 62.3% | 29 | 47.5% | 0.102 |
| III+IV | 23 | 37.7% | 32 | 52.5% |  |
| pTNM stage* |  |  |  |  |  |
| I+II | 27 | 44.3% | 16 | 26.2% | **0.037** |
| III+IVa | 34 | 55.7% | 45 | 73.8% |  |
| Regional lymph node metastasis* | | |  |  |  |
| Negative | 54 | 88.5% | 34 | 55.7% | **<0.001** |
| Positive | 7 | 11.5% | 27 | 44.3% |  |

p <0.05 was considered statistically significant.

* Chi-square test or Fisher exact.

Abbreviations: HBsAg, hepatitis B s-antigen; HBcAb, hepatitis B c-antibody; ALT, alanine aminotransferase; Tbil, total bilirubin, CA19-9, carbohydrate antigen 19-9; pTNM, pathological tumor, lymph node, metastasis classification.

**Table 2. Univariate and multivariate analysis of factors associated with survival and recurrence in Cohort 1 (n=122)**

|  | Survival^a^ | | | | | | |  | Recurrence^a^ | | | | | | |
| --- | --- | --- | --- | --- | --- | --- | --- | --- | --- | --- | --- | --- | --- | --- | --- |
| Variables | univariate analysis | | |  | multivariate analysis | | |  | univariate analysis | | |  | multivariate analysis | | |
|  | HR | 95% CI | P value |  | HR | 95% CI | P value |  | HR | 95% CI | P value |  | HR | 95% CI | P value |
| Gender (male vs female) | 1.142 | 0.684-1.905 | 0.611 |  |  |  |  |  | 1.472 | 0.844-2.565 | 0.173 |  |  |  |  |
| Age (＞50 vs ≤50) | 1.399 | 0.711-2.754 | 0.331 |  |  |  | NA |  | 1.309 | 0.637-2.690 | 0.464 |  |  |  | NA |
| HBsAg (negative vs positive) | 0.898 | 0.523-1.541 | 0.695 |  |  |  | NA |  | 1.291 | 0.731-2.279 | 0.378 |  |  |  | NA |
| HBcAb (negative vs positive) | 0.966 | 0.570-1.636 | 0.898 |  |  |  | NA |  | 1.169 | 0.640-2.136 | 0.612 |  |  |  | NA |
| CA19-9 (＞37U/ml vs ≤37U/ml) | 2.035 | 1.205-3.436 | **0.008** |  | 2.412 | 1.413-4.116 | **0.001** |  | 1.073 | 0.618-1.862 | 0.802 |  |  |  | NA |
| Cirrhosis (yes vs no) | 0.884 | 0.511-1.531 | 0.661 |  |  |  | NA |  | 1.228 | 0.691-2.183 | 0.485 |  |  |  | NA |
| Tumor size (＞5cm vs ≤5cm) | 1.064 | 0.644-1.757 | 0.810 |  |  |  | NA |  | 1.217 | 0.699-2.119 | 0.487 |  |  |  | NA |
| Tumor number (solitary vs multiple) | 1.423 | 0.612-3.310 | 0.412 |  |  |  | NA |  | 1.132 | 0.407-3.145 | 0.812 |  |  |  | NA |
| Tumor thrombosis (yes vs no) | 1.529 | 0.828-2.823 | 0.175 |  |  |  | NA |  | 1.580 | 0.806-3.096 | 0.183 |  |  |  | NA |
| Tumor capsule (yes vs no） | 1.194 | 0.589-2.422 | 0.623 |  |  |  | NA |  | 0.974 | 0.416-2.284 | 0.952 |  |  |  | NA |
| Regional lymph node metastasis(yes vs no) | 1.911 | 1.144-3.194 | **0.013** |  |  |  | NS |  | 1.914 | 1.082-3.387 | **0.026** |  |  |  | NS |
| Tumor differentiation (III+IV vs I+II) | 2.918 | 1.738-4.901 | **<0.001** |  | 2.605 | 1.530-4.433 | **<0.001** |  | 3.187 | 1.795-5.658 | **<0.001** |  | 3.178 | 1.774-5.692 | **<0.001** |
| TNM stage (III+IVa vs I+II) | 2.253 | 1.241-4.091 | **0.008** |  |  |  | NS |  | 1.635 | 0.892-2.997 | 0.112 |  |  |  | NA |
| OPN expression (low vs high) | 2.266 | 1.337-3.843 | **0.002** |  | 1.862 | 1.057-3.279 | **0.031** |  | 2.470 | 1.376-4.434 | **0.002** |  | 1.970 | 1.064-3.647 | **0.031** |

Abbreviations: HBsAg, hepatitis B s-antigen; HBcAb, hepatitis C c-antibody; Tbil, total bilirubin; CA19-9, carbohydrate antigen 19-9; pTNM, pathological tumor, lymph node, metastasis classification; NA, not adopted; NS, not significant.

a Cox proportional hazards regression.

**Table 3. Comparison of clinicopathologic profiles between low and high OPN or β-Catenin expression in ICC patients from Cohort 2 (n=180)**

| Variables | OPN (N=180) | | | | |  | β-Catenin (N=180) | | | | |
| --- | --- | --- | --- | --- | --- | --- | --- | --- | --- | --- | --- |
|  | Low (N=90) | | High (N=90) | | P |  | Low (N=90) | | High (N=90) | | P |
|  | No. of patients | % | No. of patients | % |  |  | No. of patients | % | No. of patients | % |  |
| Gender^*^ |  |  |  |  |  |  |  |  |  |  |  |
| Female | 38 | 42.2% | 34 | 37.8% | 0.543 |  | 34 | 37.8% | 38 | 42.2% | 0.543 |
| Male | 52 | 57.8% | 56 | 62.2% |  |  | 56 | 62.2% | 52 | 57.8% |  |
| Age^*^ |  |  |  |  |  |  |  |  |  |  |  |
| ≥50 | 71 | 78.9% | 70 | 77.8% | 0.856 |  | 75 | 83.3% | 66 | 73.3% | 0.103 |
| ＜50 | 19 | 21.1% | 20 | 22.2% |  |  | 15 | 16.7% | 24 | 26.7% |  |
| HBsAg^*^ |  |  |  |  |  |  |  |  |  |  |  |
| Negative | 58 | 64.4% | 66 | 73.3% | 0.198 |  | 57 | 63.3% | 67 | 74.4% | 0.107 |
| Positive | 32 | 35.6% | 24 | 26.7% |  |  | 33 | 36.7% | 23 | 25.6% |  |
| HBcAb^*^ |  |  |  |  |  |  |  |  |  |  |  |
| Negative | 31 | 34.4% | 30 | 33.3% | 0.875 |  | 27 | 30.0% | 34 | 37.8% | 0.270 |
| Positive | 59 | 65.6% | 60 | 66.7% |  |  | 63 | 70.0% | 56 | 62.2% |  |
| Cirrhosis^*^ |  |  |  |  |  |  |  |  |  |  |  |
| No | 62 | 68.9% | 67 | 74.4% | 0.408 |  | 61 | 67.8% | 68 | 75.6% | 0.247 |
| Yes | 28 | 31.1% | 23 | 25.6% |  |  | 29 | 32.2% | 22 | 24.4% |  |
| ALT (U/L) ^*^ |  |  |  |  |  |  |  |  |  |  |  |
| ≤ 75 | 61 | 67.8% | 74 | 82.2% | 0.025 |  | 62 | 68.9% | 73 | 81.1% | 0.058 |
| ＞75 | 29 | 32.2% | 16 | 17.8% |  |  | 28 | 31.1% | 17 | 18.9% |  |
| Tbil (μmol/dl) ^*^ | |  |  |  |  |  |  |  |  |  |  |
| ≤ 17.1 | 56 | 62.2% | 67 | 74.4% | 0.078 |  | 61 | 67.8% | 62 | 68.9% | 0.873 |
| ＞17.1 | 34 | 37.8% | 23 | 25.6% |  |  | 29 | 32.2% | 28 | 31.1% |  |
| GGT (U/L) | |  |  |  |  |  |  |  |  |  |  |
| ≤ 60 | 53 | 59.9% | 35 | 38.9% | **0.007** |  | 49 | 54.4% | 39 | 43.3% | 0.136 |
| ＞60 | 37 | 41.1% | 55 | 61.1% |  |  | 41 | 45.6% | 51 | 56.7% |  |
| CA19-9 (U/ml) ^*^ | |  |  |  |  |  |  |  |  |  |  |
| ≤ 37 | 44 | 48.9% | 38 | 42.2% | 0.369 |  | 46 | 51.1% | 36 | 40.0% | 0.134 |
| ＞37 | 46 | 51.1% | 52 | 57.8% |  |  | 44 | 48.9% | 54 | 60.0% |  |
| Tumor size (cm) ^*^ | |  |  |  |  |  |  |  |  |  |  |
| ≤5 | 48 | 53.3% | 38 | 42.2% | 0.136 |  | 40 | 44.4% | 46 | 51.1% | 0.371 |
| ＞5 | 42 | 46.7% | 52 | 57.8% |  |  | 50 | 55.6% | 44 | 48.9% |  |
| Tumor number^*^ | |  |  |  |  |  |  |  |  |  |  |
| Single | 84 | 93.3% | 84 | 93.3% | 1.000 |  | 85 | 94.4% | 83 | 92.2% | 0.767 |
| Multiple | 6 | 6.7% | 6 | 6.7% |  |  | 5 | 5.6% | 7 | 7.8% |  |
| Tumor encapsulation^*^ | |  |  |  |  |  |  |  |  |  |  |
| None | 74 | 82.2% | 83 | 92.2% | 0.072 |  | 80 | 88.9% | 77 | 85.6% | 0.503 |
| Complete | 16 | 17.8% | 7 | 7.8% |  |  | 10 | 11.1% | 13 | 14.4% |  |
| Tumor thrombus^*^ | |  |  |  |  |  |  |  |  |  |  |
| No | 78 | 86.7% | 78 | 86.7% | 1.000 |  | 83 | 92.2% | 73 | 81.1% | **0.047** |
| Yes | 12 | 13.3% | 12 | 13.3% |  |  | 7 | 7.8% | 17 | 18.9% |  |
| Tumor differentiation^*^ | |  |  |  |  |  |  |  |  |  |  |
| I+II | 57 | 63.3% | 46 | 51.1% | 0.097 |  | 58 | 64.4% | 45 | 50.0% | 0.050 |
| III+IV | 33 | 36.7% | 44 | 48.9% |  |  | 32 | 35.6% | 45 | 50.0% |  |
| pTNM stage^*^ | |  |  |  |  |  |  |  |  |  |  |
| I+II | 40 | 44.4% | 25 | 27.8% | **0.020** |  | 37 | 41.1% | 28 | 31.1% | 0.163 |
| III+IVa | 50 | 55.6% | 65 | 72.2% |  |  | 53 | 58.9% | 62 | 68.9% |  |
| Regional lymph node metastasis^*^ | | | |  |  |  |  |  |  |  |  |
| Negative | 78 | 86.7% | 64 | 71.1% | **0.011** |  | 76 | 84.4% | 66 | 73.3% | 0.068 |
| Positive | 12 | 13.3% | 26 | 28.9% |  |  | 14 | 15.6% | 24 | 26.7% |  |

p <0.05 was considered statistically significant.

* Chi-square test or Fisher exact.

Abbreviations: HBsAg, hepatitis B s-antigen; HBcAb, hepatitis B c-antibody; ALT, alanine aminotransferase; Tbil, total bilirubin, CA19-9, carbohydrate antigen 19-9; pTNM, pathological tumor, lymph node, metastasis classification.

**Table 4. Univariate analysis of factors associated with survival**

**and recurrence in Cohort 2 (n=180)**

| Variables | Survival^a^ | | |  |  | Recurrence^a^ | | |  |
| --- | --- | --- | --- | --- | --- | --- | --- | --- | --- |
|  | HR | 95% CI | P value |  |  | HR | 95% CI | P value |  |
| Gender (male vs female) | 1.037 | 0.677-1.590 | 0.866 |  |  | 1.213 | 0.770-1.910 | 0.404 |  |
| Age (＞50 vs ≤50) | 0.907 | 0.555-1.481 | 0.696 |  |  | 0.975 | 0.575-1.655 | 0.925 |  |
| HBsAg (negative vs positive) | 0.815 | 0.512-1.295 | 0.386 |  |  | 1.185 | 0.741-1.896 | 0.479 |  |
| HBcAb (negative vs positive) | 0.824 | 0.538-1.263 | 0.375 |  |  | 1.023 | 0.637-1.644 | 0.924 |  |
| CA19-9 (＞37U/ml vs ≤37U/ml) | 2.241 | 1.441-3.487 | **<0.001** |  |  | 1.103 | 0.703-1.731 | 0.670 |  |
| Cirrhosis (yes vs no) | 0.751 | 0.463-1.218 | 0.246 |  |  | 0.812 | 0.487-1.353 | 0.424 |  |
| Tumor size (＞5cm vs ≤5cm) | 1.053 | 0.693-1.601 | 0.808 |  |  | 1.383 | 0.877-2.181 | 0.163 |  |
| Tumor number (solitary vs multiple) | 0.848 | 0.343-2.094 | 0.720 |  |  | 0.726 | 0.265-1.988 | 0.533 |  |
| Tumor thrombosis (yes vs no) | 1.509 | 0.863-2.638 | 0.149 |  |  | 1.419 | 0.764-2.635 | 0.268 |  |
| Tumor capsule (yes vs no） | 0.976 | 0.528-1.805 | 0.938 |  |  | 0.895 | 0.446-1.795 | 0.754 |  |
| Regional lymph node metastasis(yes vs no) | 1.703 | 1.057-2.744 | **0.029** |  |  | 1.289 | 0.751-2.212 | 0.358 |  |
| Tumor differentiation (III+IV vs I+II) | 2.112 | 1.387-3.218 | **<0.001** |  |  | 2.333 | 1.481-3.675 | **<0.001** |  |
| pTNM stage (III+IVa vs I+II) | 2.586 | 1.554-4.301 | **<0.001** |  |  | 2.373 | 1.395-4.039 | **0.001** |  |
| OPN expression (high vs low) | 2.441 | 1.569-3.798 | **<0.001** |  |  | 2.908 | 1.788-4.728 | **<0.001** |  |
| β-catenin expression (high vs low) | 2.577 | 1.655-4.012 | **<0.001** |  |  | 2.212 | 1.390-3.522 | **0.001** |  |
| Combine OPN with β-catenin expression^b^ |  |  | **<0.001** |  |  |  |  | **<0.001** |  |
| II vs I | 2.038 | 1.105-3.759 | **0.023** |  |  | 1.919 | 1.001-3.679 | **0.005** |  |
| III vs I | 3.872 | 2.211-6.780 | **<0.001** |  |  | 3.776 | 2.102-6.783 | **<0.001** |  |

Abbreviations: HBsAg, hepatitis B s-antigen; HBcAb, hepatitis C c-antibody; Tbil, total bilirubin; CA19-9, carbohydrate antigen 19-9; pTNM, pathological tumor, lymph node, metastasis classification; NA, not adopted; NS, not significant.

a Cox proportional hazards regression.

b The cohort was classified into three groups according to their OPN and β-Catenin expression: group I (n=53), both low expression; group II (n=70), high OPN but low β-Catenin expression or low β-Catenin but high OPN expression; and group III (n=53), both high expression.

**Table 5. Multivariate analysis of factors associated with survival**

**and recurrence in Cohort 2 (n=180)**

| Variables |  | Survival^a^ | | |  |  | Recurrence^a^ | | |
| --- | --- | --- | --- | --- | --- | --- | --- | --- | --- |
|  |  | HR | 95% CI | P value |  |  | HR | 95% CI | P value |
| **multivariate analysis** |  |  |  |  |  |  |  |  |  |
| CA19-9 (＞37U/ml vs ≤37U/ml) |  | 2.074 | 1.330-3.235 | **0.001** |  |  |  |  | NA |
| Regional lymph node metastasis(yes vs no) |  | 1.222 | 0.741-2.014 | 0.432 |  |  |  |  | NA |
| Tumor differentiation (III+IV vs I+II) |  | 1.730 | 1.129-2.652 | **0.012** |  |  | 1.986 | 1.257-3.138 | **0.003** |
| pTNM stage (III+IVa vs I+II) |  | 2.115 | 1.234-3.627 | **0.006** |  |  | 2.125 | 1.241-3.642 | **0.006** |
| OPN expression (high vs low) |  | 1.607 | 1.007-2.564 | **0.047** |  |  | 2.128 | 1.259-3.597 | **0.005** |
| β-catenin expression (high vs low) |  | 2.044 | 1.287-3.245 | **0.002** |  |  | 1.564 | 0.949-2.577 | 0.079 |
| **multivariate analysis** |  |  |  |  |  |  |  |  |  |
| CA19-9 (＞37U/ml vs ≤37U/ml) |  | 2.132 | 1.359-3.343 | **0.001** |  |  |  |  | NA |
| Regional lymph node metastasis(yes vs no) |  | 1.256 | 0.760-2.074 | 0.374 |  |  |  |  | NA |
| Tumor differentiation (III+IV vs I+II) |  | 1.767 | 1.148-2.721 | **0.010** |  |  | 1.999 | 1.264-3.162 | **0.003** |
| pTNM stage (III+IVa vs I+II) |  | 2.033 | 1.191-3.471 | **0.009** |  |  | 2.175 | 1.273-3.717 | **0.004** |
| Combine OPN with β-catenin expression |  |  |  | **<0.001** |  |  |  |  | **<0.001** |
| II vs I |  | 1.461 | 0.780-2.737 | 0.236 |  |  | 1.683 | 0.874-3.244 | 0.120 |
| III vs I |  | 3.846 | 1.753-5.470 | **<0.001** |  |  | 3.240 | 1.793-5.857 | **<0.001** |

a Cox proportional hazards regression.

**Table 6. Antibodies for western blot, immunohistochemistry, co-immunoprecipitation and immunofluorescence**

| Antibody | WB | IHC | Co-IP | IF | Specificity | Company | Number |
| --- | --- | --- | --- | --- | --- | --- | --- |
| OPN | + | + | + | + | monoclonal | Abcam | 69498 |
| β-catenin | + | + | + | + | monoclonal | CST | 8480 |
| Phospho-β-catenin(S675) | + | - | + | - | polyclonal | CST | 9567 |
| Anti-Phospho - (Ser/Thr) | + | - | + | + | monoclonal | Abcam | 17464 |
| Phospho-MEK1/2 (Ser217/221) | + | - | + | - | monoclonal | CST | 3958 |
| p44/42MAPK (Erk1/2) | + | + | + | + | monoclonal | CST | 4695 |
| MAPK1(p42) | + | + | - | - | monoclonal | Sigma | HPA030069 |
| MAPK1(p42) | + | + | + | + | monoclonal | Abcam | 32081 |
| Phospho-p44/42 MAPK(Erk1/2) (Thr202/Tyr204) | + | + | + | + | monoclonal | CST | 4370 |
| β-Tubulin | + | + | - | + | monoclonal | CST | 2128 |
| HA-tag | + | + | + | + | monoclonal | CST | 3724 |
| Flag-tag | + | + | + | + | monoclonal | CST | 14793 |
| CyclinD1 | + | + | - | - | monoclonal | CST | 2978 |
| c-Myc | + | + | + | + | monoclonal | Abcam | 32072 |
| Prox1 | + | + | - | - | monoclonal | CST | 14963 |

**Supplementary figure legends**

**Supplementary Figure 1. OPN downregulation decreases the growth and metastasis of ICC cells.** (A-C) Confirmation of OPN knockdown (Scr, shOPN-1, shOPN-2, shOPN-3) in HuCCT1 cells (A) and CCLP1 (B) cells used quantitative real-time PCR and immunoblotting (IB). shOPN-3 showed the best efficiency of OPN knockdown. Then OPN expression was rescued with OPN re-expression plasmid (shRES) (C). (D) Statistical data of colony formation in HuCCT1(left) and CCLP1(right) cell lines (Scr, shOPN-3, shRES). (E-F) Downregulation of OPN significant inhibited migration (E) and invasion (F) of HuCCT1 (left) and CCLP1 (right) cells. Those phenotypes can be counteracted by shRES. All assays were performed in triplicate wells and repeated three times for each cell line, scale bar = 200 μm.

**Supplementary Figure 2.** **Effects of OPN on ICC progression.** ICC cells (RBE, HCCC9810, HuH-28) were transfected with empty vector and OPN. Comparison of control group, overexpression of OPN remarkably promoted the proliferation (A), colony formation (B), migration (C) and invasion (D) of RBE cells (left), HCCC9810 cells (middle) and HuH-28 cells (right), scale bar = 200 μm.

**Supplementary Figure 3.** **OPN is demonstrated to interact with β-Catenin.** (A) Mass spectrometry (MS) and immunoprecipitation were used to analyze OPN-associated proteins. Empty vector or Flag-OPN was stably expressed in CCLP1 cells, then the cell lysates were subjected to immunoaffinity purification and the purified product was identified by SDS-PAGE and silver staining. Identified peptides of β-Catenin are shown. (B) Colocalization of OPN (red) and β-Catenin (green) was determined by immunofluorescence analysis in RBE cells, scale bar = 20 μm. (C-D) IF staining of OPN (red) and β-Catenin (green) in ICC tumor tissues, scale bar = 50 μm. (E) Representative IHC staining results of OPN and β-Catenin, Case 1: OPN^high^ β-Catenin^high^, Case 2: OPN^low^ β-Catenin ^low^, scale bar = 50 μm.

**Supplementary Figure 4. OPN regulates the Wnt/β-Catenin signaling pathway.** (A) Knockdown of OPN in HuCCT1 cells can remarkably inhibit the mRNA expression of cyclinD1, c-Myc and Prox1. (B) Overexpressing OPN in HCCC9810 significantly activated the downstream of Wnt signaling pathway.

**Supplementary Figure 5. β-Catenin can rescue the growth and metastasis inhibition induced by downregulation of OPN.** (A-B) Knockdown of OPN significantly suppressed the colony formation (A) and migration (B), which could be restored by β-Catenin overexpression in CCLP1 and HuCCT1 cells. (C) The dynamic change of tumor volume in subcutaneous tumor model was examined using the equation *V* =*ab*^2^/2(*a,* length; *b*, width). (D) Nude mice were implanted with RBE cell lines (empty vector, Flag-OPN or Flag-OPN plus Siβ-Catenin) into the intraperitoneal cavity and were killed 6 weeks after injection, liver metastasis nodes were counted under microscope. (E) Nuclear and cytosol fractionation assay were performed using cell lysates of HCCC9810 with empty vector or OPN over-expressed, and β-Catenin expression was analyzed by western blot. (F) RBE cells, with β-Catenin wild type or mutants (S191A or S605A) stably over-expressed, were transfect with OPN or empty vector then cell lysates were subjected to IP and IB to detect the Serine phosphorylation level of β-Catenin.

**Supplementary Figure 6. Knockdown of OPN and MAPK1 or inhibiting the activity of MAPK1 can reduce the phosphorylation of S675.** (A) OPN upregulation activatd the MEK/MAPK1 pathway and promote the protein level of β-Catenin in RBE cells, which could be inhibit by U0126 treatment. (B) Immunoprecipitation (IP) and Immunoblotting(IB) assays showed the effect of OPN and MAPK1 overexpression in phosphorylation of β-Catenin at S675 in RBE cells. (C) Immunoblotting (IB) verification of endogenous OPN or MAPK1 knockdown in CCLP1 cells. OPN and MAPK1was detected by anti-OPN or anti-MAPK1 mAb (left). Co-IP and IB was performed to analyze β-Catenin S675 phosphorylation in RBE cells with endogenous OPN or MAPK1 knockdown (right). (D) Western blot was performed to determine the expression of OPN, β-Catenin, MAPK1, pMAPK1, pMEK, Prox1, c-Myc between HuCCT1-Scr group and HuCCT1-shOPN group in the xenograft tumor tissues antibodies specific for each protein. (E) The inhibition of MEK/MAPK1 pathway and Wnt/β-Catenin pathway were examined by IHC staining in two xenograft groups (HuCCT1-Scr and HuCCT1-shOPN), Scale bar, 200 uM.

**Supplementary Figure 7.** **OPN activates MAPK1 and the phosphorylation of Ser675 of β-Catenin to promote the growth and invasion of ICC.** (A-B) Overexpression of OPN significantly increased colony formation (A) and invasion abilities of ICC cells (B),then those cell phenotypes, which were counteracted by wild re-expression with β-Catenin-WT but not S675A orβ-Catenin-WT plus U0126 treatment.

**Supplementary Figure 8.** **The combination of OPN and β-Catenin levels was assessed by ROC curve analysis.** Larger areas under curve indicate more predictive power of the variable. The combination of OPN and β-Catenin levels showed the best predictive capacity compared with the other groups for overall survival (A) and recurrence (B).

**Supplementary Figure 9. Schematic model of the OPN mediated S675 phosphorylation and nucleus accumulation of β-Catenin via recruiting and activating MAPK1 in ICC.** OPN selectively interacted with β-catenin and mediated its S675 phosphorylation and nucleus accumulation through recruiting and activating MAPK1, and resulted in activation of Wnt/β-catenin pathway and metastasis of ICC.
