# Supplementary figures and images for: Osteopontin promotes metastasis of intrahepatic cholangiocarcinoma through recruiting MAPK1 and mediating Ser675 phosphorylation of β-Catenin
Source: Cell Death Dis. 2018 Feb 7;9(2):179. doi: 10.1038/s41419-017-0226-x (PMC5833342; doi:10.1038/s41419-017-0226-x)

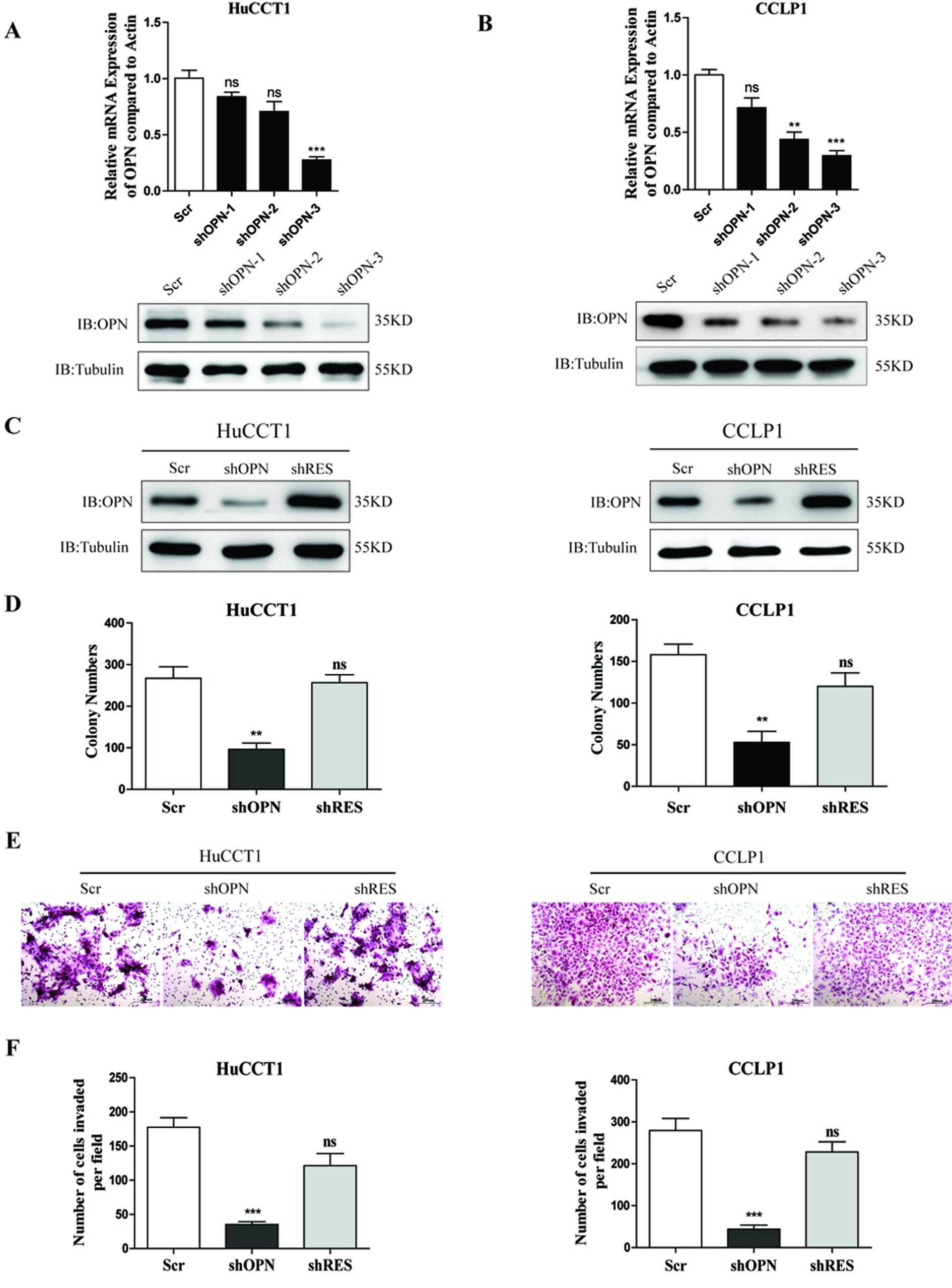

Supplement: Supplementary file 2 — Supplementary Figure1 [file 41419_2017_226_MOESM2_ESM.tif]

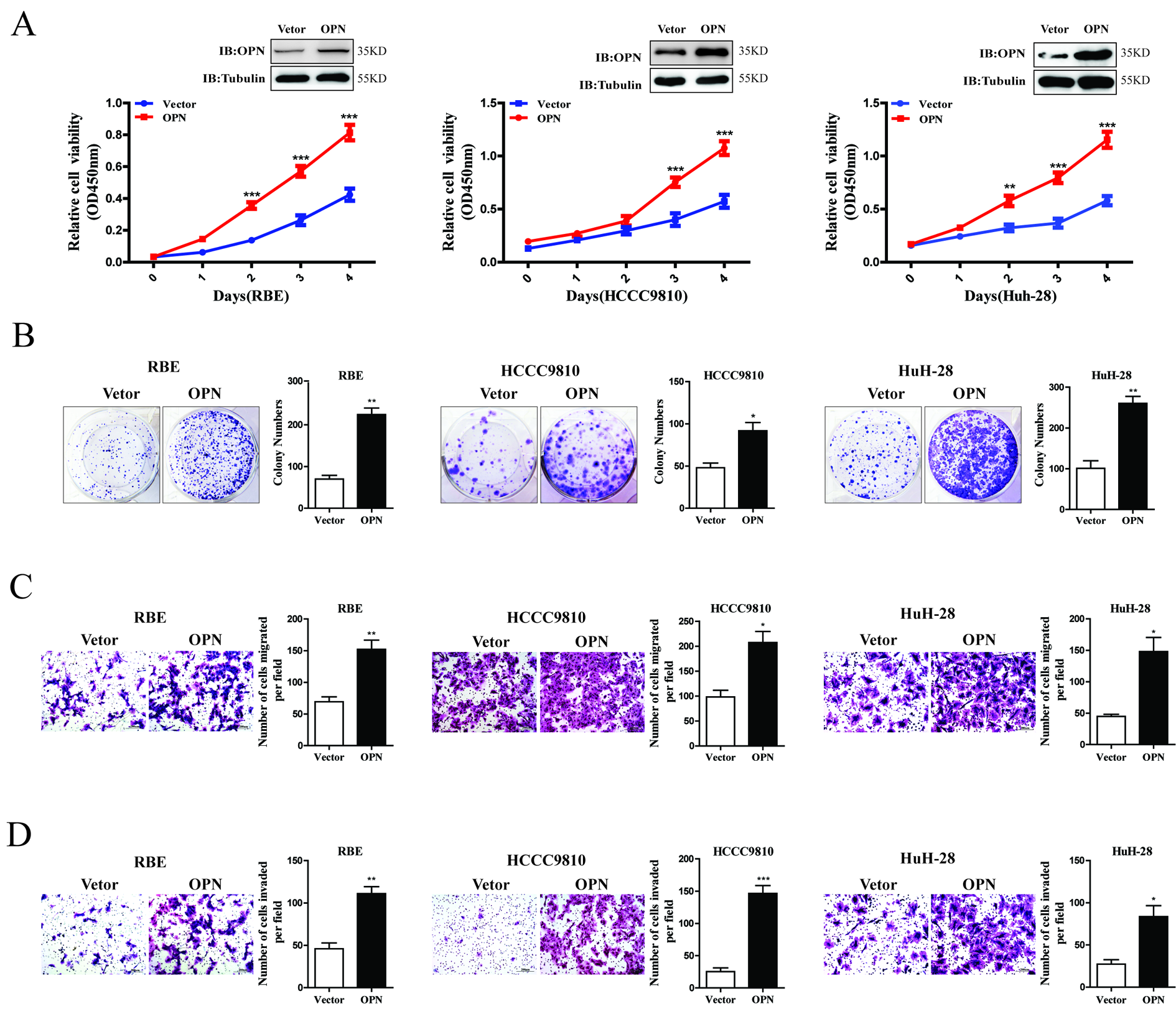

Supplement: Supplementary file 3 — Supplementary Figure2 [file 41419_2017_226_MOESM3_ESM.tif]

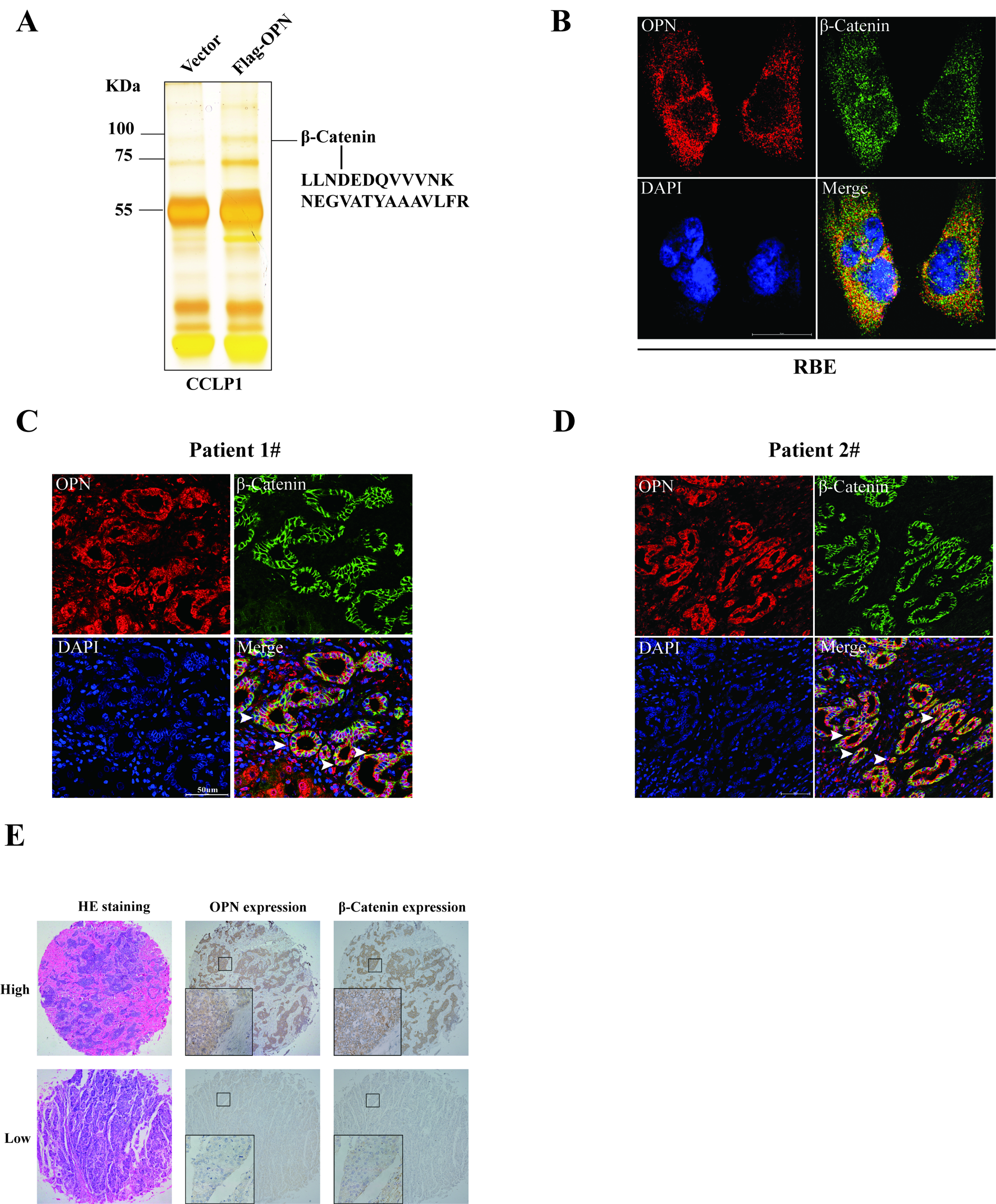

Supplement: Supplementary file 4 — Supplementary Figure3 [file 41419_2017_226_MOESM4_ESM.tif]

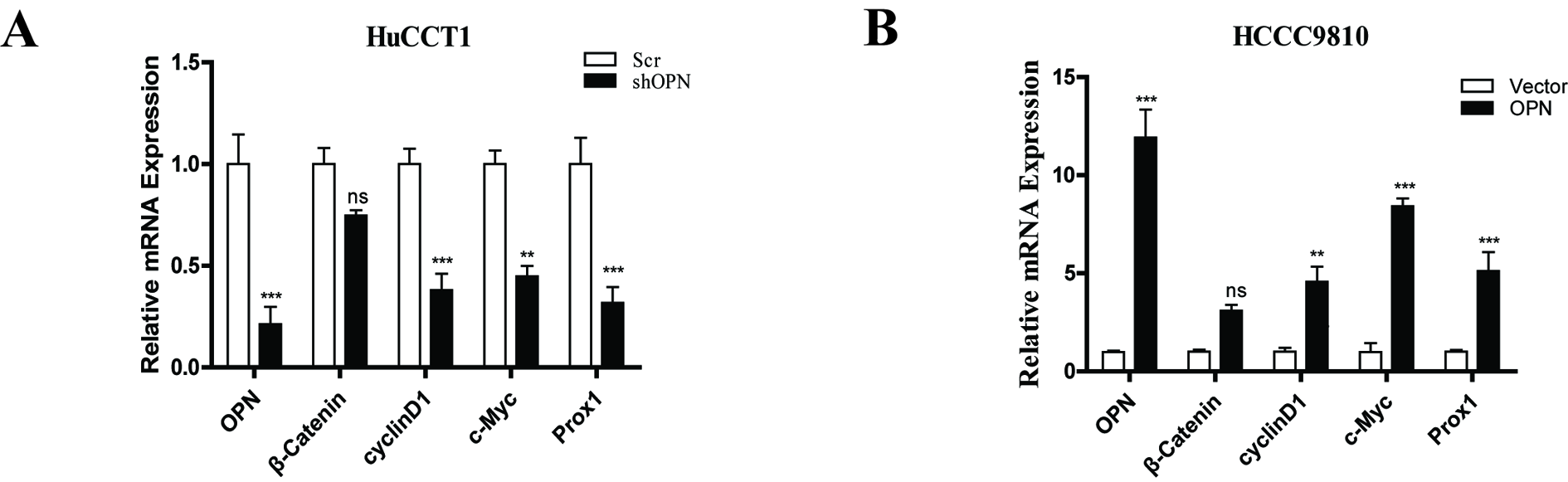

Supplement: Supplementary file 5 — Supplementary Figure4 [file 41419_2017_226_MOESM5_ESM.tif]

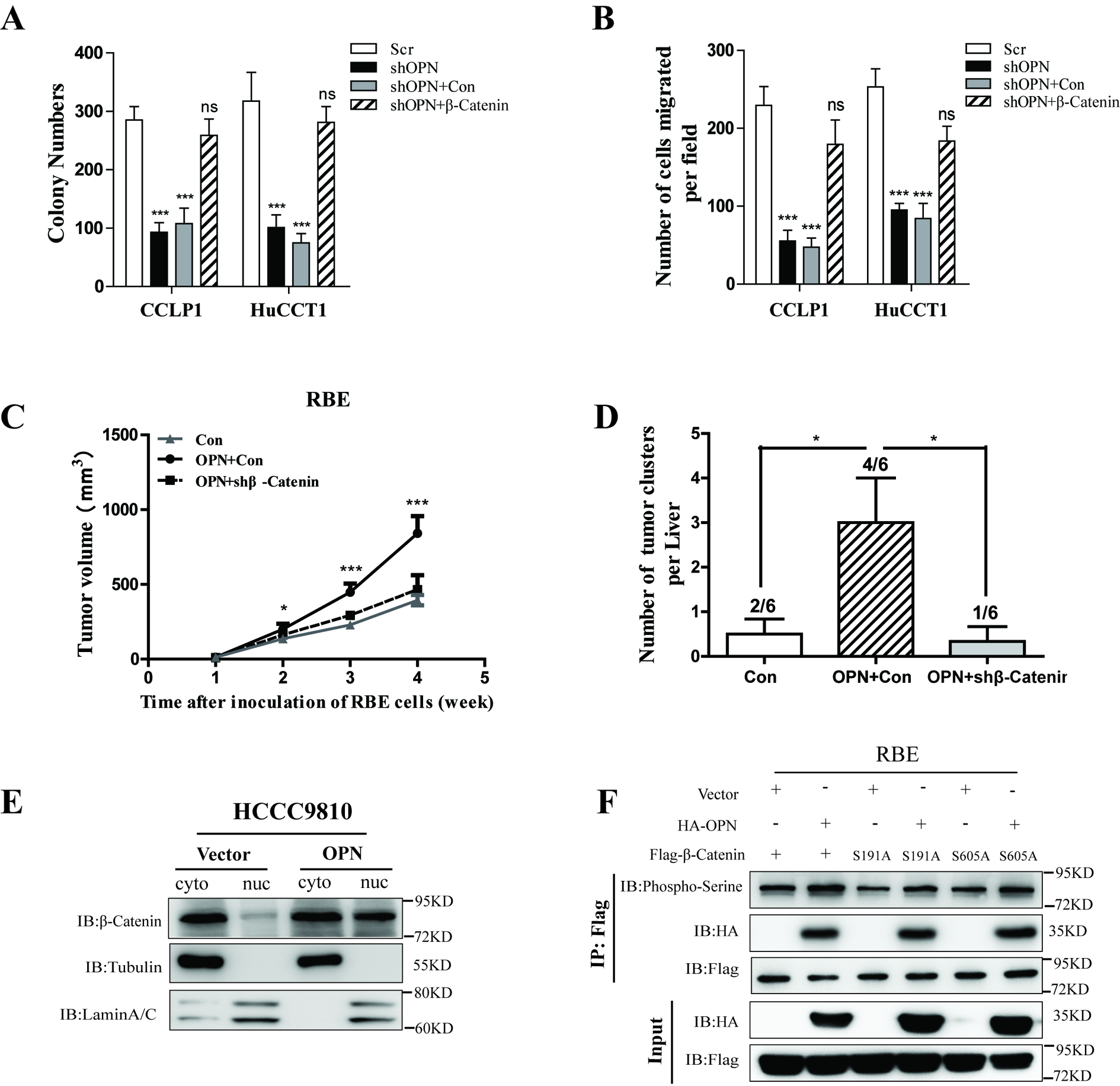

Supplement: Supplementary file 6 — Supplementary Figure5 [file 41419_2017_226_MOESM6_ESM.tif]

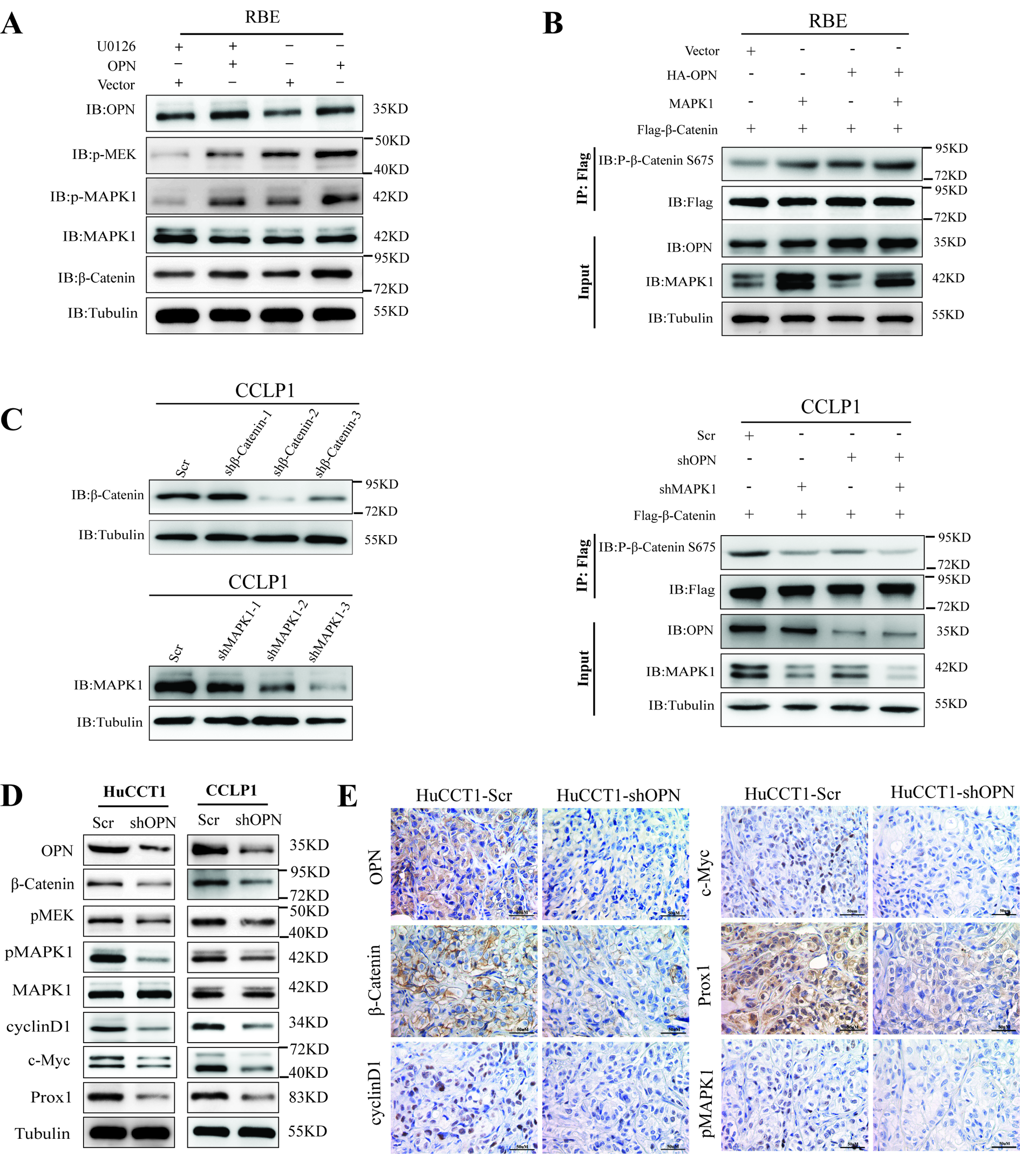

Supplement: Supplementary file 7 — Supplementary Figure6 [file 41419_2017_226_MOESM7_ESM.tif]

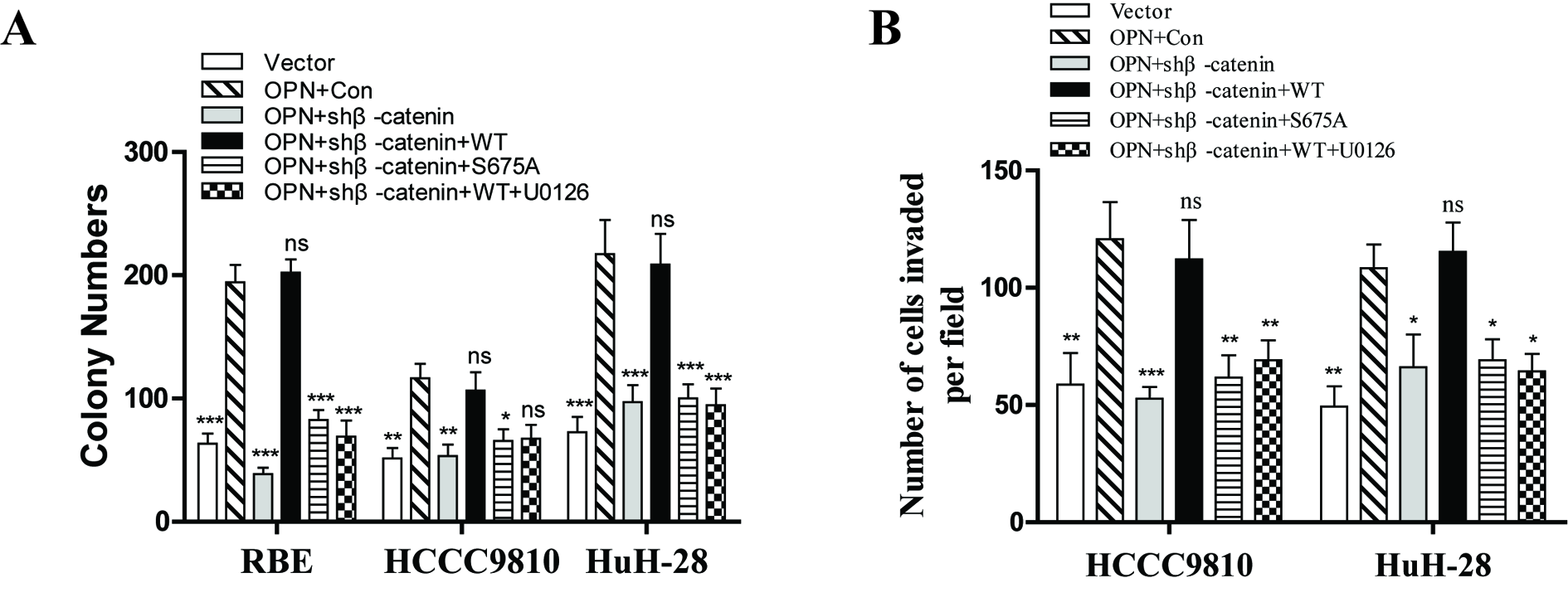

Supplement: Supplementary file 8 — Supplementary Figure7 [file 41419_2017_226_MOESM8_ESM.tif]

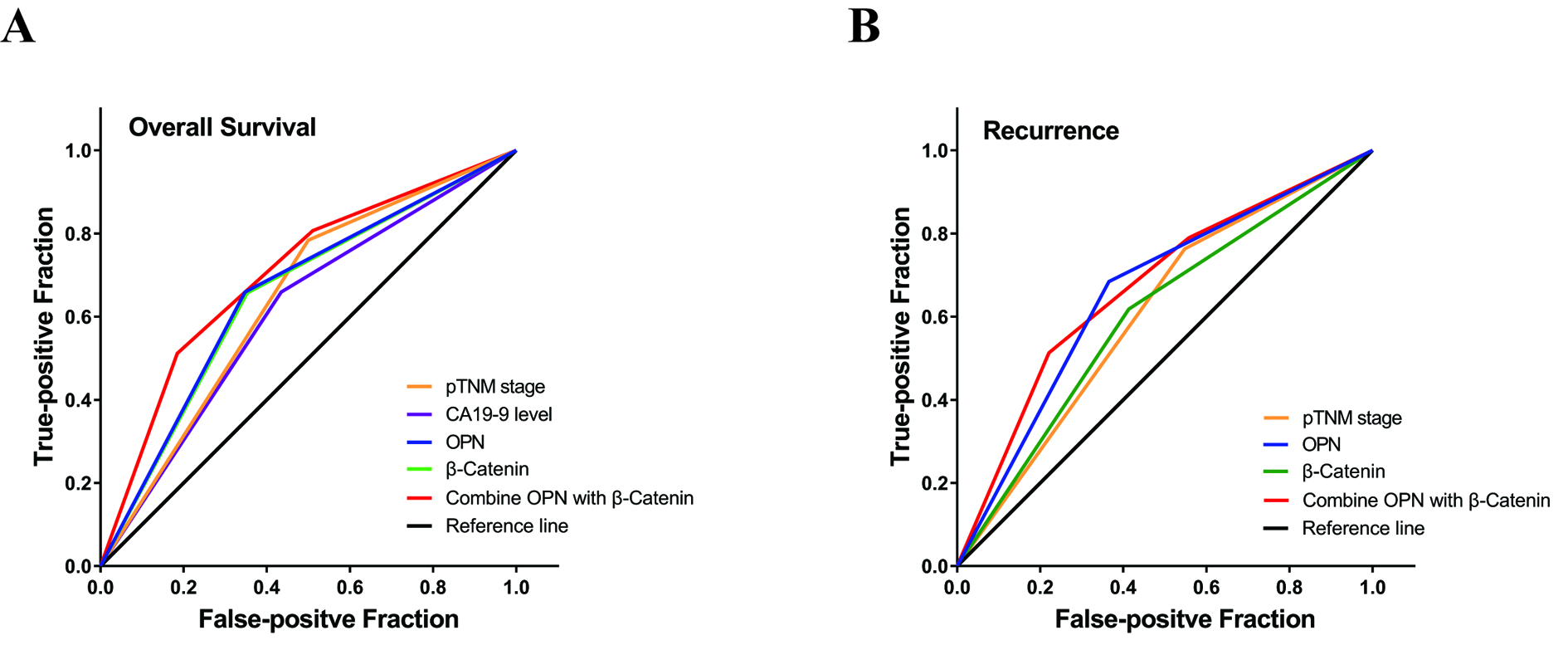

Supplement: Supplementary file 9 — Supplementary Figure8 [file 41419_2017_226_MOESM9_ESM.tif]

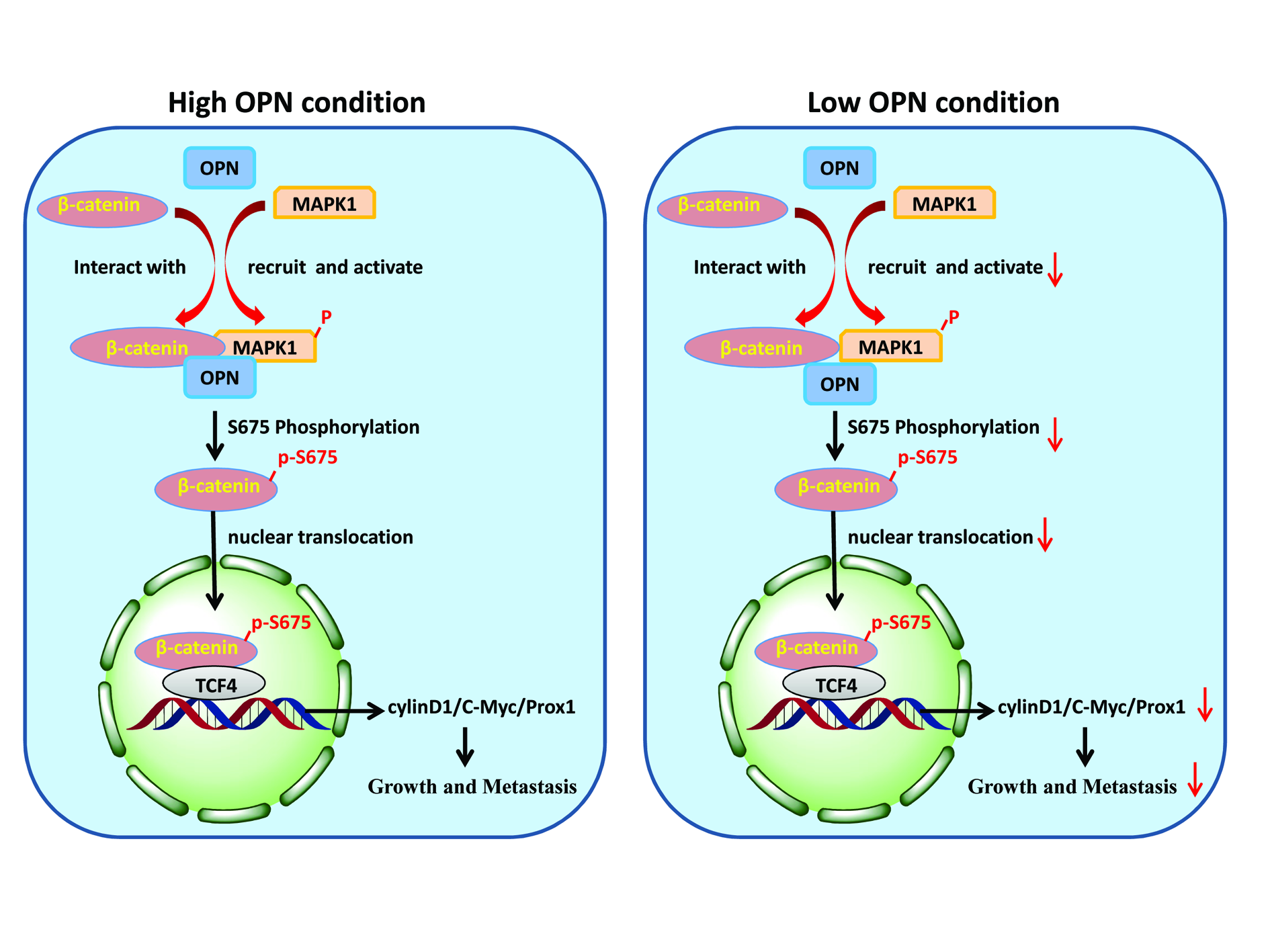

Supplement: Supplementary file 10 — Supplementary Figure 9 [file 41419_2017_226_MOESM10_ESM.tif]
